# Interplay between Inter- and Intramolecular Halogen-/Chalcogen-Bonding in Two-Molecule Aggregates Featuring Te^II^···I Secondary-Bonding Interactions and in Their Congeners

**DOI:** 10.1021/acs.jpca.6c00421

**Published:** 2026-05-05

**Authors:** Rosa M. Gomila, Antonio Frontera, Edward R. T. Tiekink

**Affiliations:** Department of Chemistry, 16745 Universitat de les Illes Balears, Crta de Valldemossa km 7.5, Palma de Mallorca 07122, Spain

## Abstract

The Cambridge Structural Database (CSD) was searched for crystals containing both tellurium­(II) and iodine, revealing ten molecular crystals featuring distinct two-molecule aggregates encompassing Te···I interactions. These supramolecular assemblies were classified into three categories based on their geometric characteristics: halogen bonds (HaB), chalcogen bonds (ChB), and Type I interactions. A systematic structural comparison with lighter congeners (substituting Te with Se, S, and I with Br, Cl, and F) highlighted the exclusive propensity of tellurium and iodine to form these specific motifs, often with limited isostructurality across the series. To provide deeper insight into the nature of these noncovalent interactions, Density Functional Theory (DFT) calculations (PBE0-D3/def2-TZVP) were conducted on five representative systems. The computational results, including Molecular Electrostatic Potential (MEP) surfaces, QTAIM, and NBO analyses, revealed that the supramolecular organization is governed by a delicate interplay between the nature of intermolecular Te···I contacts and additional Te···N, O, S, and Te contacts, intramolecular or external, which are classified as ChBs exclusively. The energetic analysis demonstrated that while some aggregates are driven by robust HaBs, others rely on the cooperative effects of weaker ChBs and auxiliary interactions, with substantial orbital charge transfer contributions confirmed by NBO analysis.

## Graphical abstract

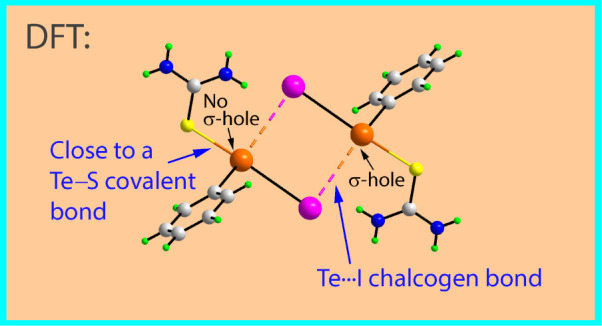

## 1 Introduction

Owing to their directionality and strength, chalcogen-bonding (ChB)^1,2^ and the more studied halogen-bonding (HaB)^3,4^ have emerged as important noncovalent interactions (NCIs) in supramolecular chemistry and have been the subject of substantial reviews. Thus, reviews/prominent studies of the impact of ChB are available: comprehensive overviews,^5^ materials science,^6,7^ catalysis,^8–10^ crystal engineering,^11^ and the role of ChB in coordination chemistry.^12^ A comprehensive review of HaB is a reference point for studies in this area,^13^ and more specialized aspects of HaB are available, relating to materials chemistry,^14^ molecule/anion recognition,^15,16^ catalysis,^17^ systematic modulation of HaB strength,^18,19^ and its relationship to chirality.^20^ Complementing the reviews focused on the nature and applications of ChB and HaB, there are authoritative reviews on the direct relationships between hydrogen-bonding and each of ChB^21^ and HaB,^22^ and recent studies have highlighted the interplay between ChB and HaB.^23–26^ The rationale for ChB and HaB derives from the well-documented σ-hole phenomenon,^27–29^ as well as the π-hole concept;^30–32^ these can also act in concert^33,34^ and simultaneously.^35^ Relevant to the present study, among the commonly accessible elements of Groups 16 and 17, σ-holes will be at their maximum for the heavier tellurium and iodine atoms, correlating with the greater polarizability of the bonds directly opposite the σ-holes. Herein, an investigation of the complementarity/competition between ChB and HaB is presented for a series of two-molecule aggregates featuring at least one Te···I interaction between the interacting molecules.

Indeed, a fascinating scenario arises when tellurium­(II) and iodine functionalities coexist within the same molecular framework or aggregate. In such systems, a complex interplay between inter- and intramolecular forces can occur. Both atoms possess the dual capacity to act as electrophiles (via their σ-holes) or nucleophiles (via their lone pairs or electron-rich belts). Consequently, the geometric arrangement of a Te···I contact dictates its classification: it may manifest as a generic Type I interaction (dispersion-dominated), a HaB (where iodine acts as the electrophile), or a ChB (where tellurium acts as the electrophile). The present study focuses on this specific interplay. Experimental structures were obtained from a systematic search of the Cambridge Structural Database (CSD)^36^ designed to identify discrete two-molecule aggregates featuring Te···I interactions. This survey yielded nine distinct structural motifs among the 10 identified aggregates. To contextualize these findings, the observed geometric preferences are compared with their lighter congeners (systematically substituting Te with Se, S, and I with Br, Cl, and F). Being published results, the interested reader is encouraged to refer to the original publications to gain more information concerning additional characterization. The authors acknowledge that many of the 10 dimeric aggregates discussed herein were determined as part of wider physicochemical studies and that often assessments of the molecular packing were scant. In the same way, there are limited systematic relationships among the 10 dimeric aggregates, as most were drawn from independent investigations. The present study focuses upon the nature of ChBs and HaBs for 10 molecules in two-molecule aggregates determined crystallographically, i.e., in situ within their respective crystalline manifolds.

HaBs involving iodine are of interest beyond crystal engineering endeavors. For instance, a recent related study explored the nature of aggregation found in aggregates featuring both tellurium­(II)/(IV) and iodine.^36^ Here, it was demonstrated that in the limited number of aggregates, tellurium­(II) was more likely to form Te···I interactions than tellurium­(IV); notably, all interactions are classified as ChBs. Herein, a systematic search of the Cambridge Structural Database (CSD)^37^ was conducted to identify discrete two-molecule aggregates featuring Te···I interactions. Crucially, Density Functional Theory (DFT) calculations were employed to unravel the electronic nature of these contacts. This computational analysis assesses the relative contributions of electrostatic and orbital effects, providing a deeper understanding of the noncovalent adducts identified in this work.

## 2 Methods

The Cambridge Structural Database (CSD; version 6.00 with the August 2025 update)^37^ was searched using ConQuest (version 2025.2.0)^38^ for all crystals containing both tellurium­(II) and iodine, with Te···I separations ≤4.44 Å, this being sum of the van der Waals radii of tellurium (2.06 Å) and iodine (1.98 Å)^39^ plus 10%. Search “hits” had to satisfy the following criteria: single-crystal data, three-dimensional coordinates available, no errors, no disorder, nonpolymeric, and charge-neutral. Manual sorting revealed 10 crystals, i.e., **1**–**10**,^40–49^ presenting two-molecule aggregates with Te···I interactions between the constituent molecules—these form the basis of the present investigation. Hydrogen atoms for **1** were included in the model employing Mercury.^50^ The analysis of **1**–**10** was conducted with PLATON^39^ and DIAMOND,^51^ the latter employed to generate original crystallographic diagrams.

Computational chemistry calculations were carried out with the Gaussian-16 program,^52^ using the crystallographic coordinates available in the respective CIFs as input for the evaluation of the supramolecular assemblies. The level of theory used for the calculations was PBE0-D3 ^54^/def2-TZVP.^55,56^ For heavier elements, such as tellurium and iodine, this basis set includes effective core potentials (ECPs) and takes into consideration relativistic effects for the inner electrons.^56^ This combination of method and basis set has been previously used by us^57^ and others^58^ to analyze σ-hole interactions, including ChBs involving tellurium.^59^ The MEP surface plots were generated using the wave function obtained at the same level of theory and 0.001 au isosurfaces. The topological analysis of the electron density was carried out according to the quantum theory of atoms in molecules (QTAIM) method proposed by Bader^60,61^ and represented using the AIMAll program.^62^ The NBO analysis^63^ was performed using the same level of theory and the NBO7.0 program.^64^ The NBOs and charge density plots were represented using VMD software.^61^

## 3 Results and Discussion

### 3.1 CSD Survey and Structural Analysis

The CSD^37^ was searched for two-molecule aggregates featuring at least one Te···I interaction between them: this search yielded 10 examples, **1**–**10**.^40–49^ The search was based on the sum of the standard van der Waals radii for tellurium and iodine, i.e., 4.04 Å, plus 10%, in recognition of the observation that significant energies of stabilization can be associated with interactions beyond the sum of conventional van der Waals radii, as demonstrated in recent studies on Hg···S spodium-bonding in the crystals of mercury dithiocarbamates, i.e., molecules of the general formula Hg­(S_2_CNRR′)_2_, R, R′ = alkyl, aryl.^65,66^ Chemical diagrams for **1**–**10** are shown in Scheme 1, and geometric parameters defining the observed ChB/HaB in the crystals of **1**–**10** are listed in Table 1. In addition, in recognition of recent studies showing that valuable insight is gained about the prevalence of HaB/ChB,^67–69^ the CSD was also searched for the lighter congeners of **1**–**10**, i.e., where Te was systematically replaced by Se, S, and O, and I by Br, Cl, and F. Descriptions, geometric parameters, and relevant diagrams for the retrieved crystals are given in the Supporting Information (SI). Finally, in instances where the tellurium and/or iodine atoms engage in further intermolecular interactions in the crystal, data and images are also included in the SI, as well as for conventional hydrogen bonding when present. In the following, the two-molecule aggregates are discussed in increasing order of the number of Te···I interactions between the constituent molecules, aggregates with no further interactions between them (**2**–**4**), molecules forming additional intramolecular interactions (**5**-–**7**), and molecules forming additional external interactions (**8**–**10**); within each category, motifs are discussed in terms of increasing Te···I separations.

**Scheme 1:**
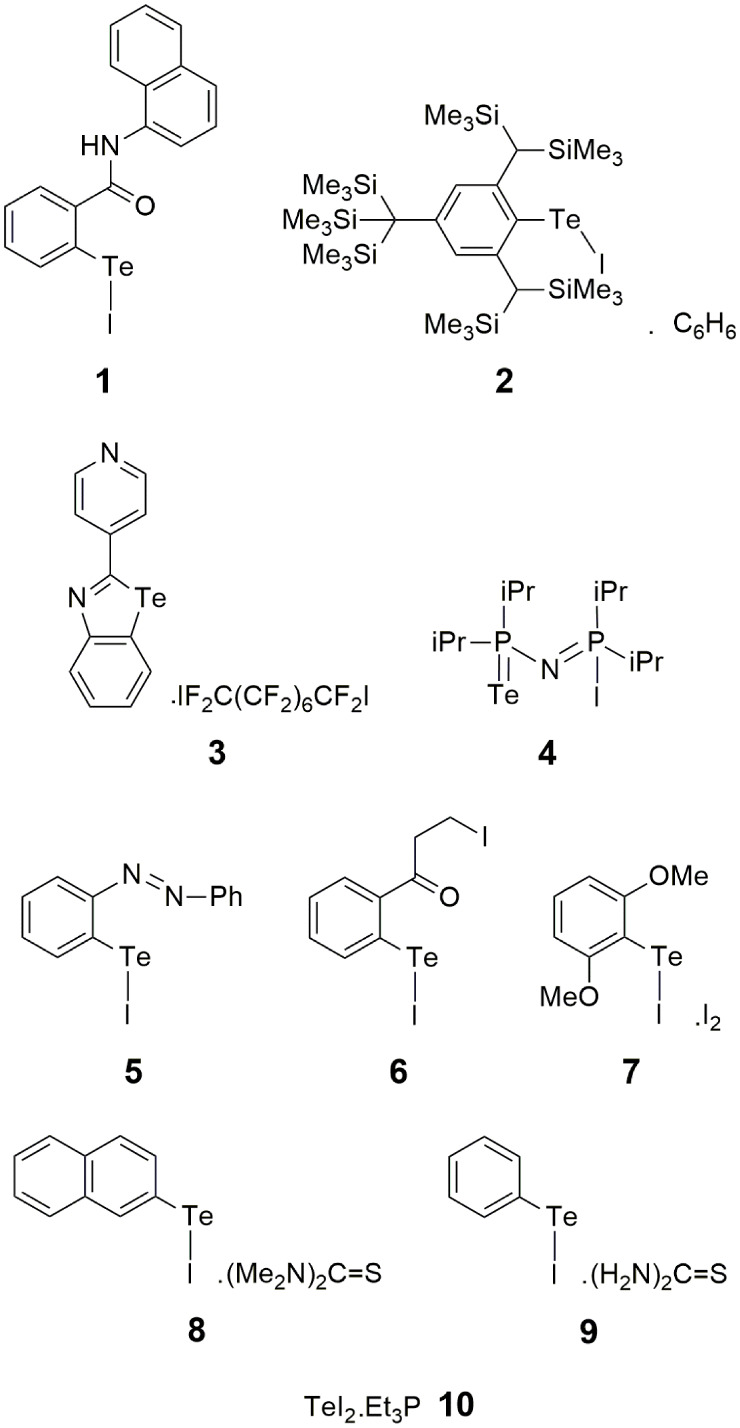
Chemical Drawings of Compounds **1**–**9** and Chemical Formula of **10**

**Table 1:** Geometric Characteristics for ChB/HaB/Type I Interactions in **1**–**10**

Aggregate	Distance (Å)	%(d/vdW)^a^	Angle (°)	NCI^b^	refcode	refs
1^c^	Te···I = 3.8454(4)	95.2	Te–I···Te = 170.313(14)	HaB	HIRRIN	^40^
	Te···O = 2.384(3)	66.6	I–Te···O = 170.71(7)	ChB		
	Te···O = 2.382(3)	66.5	I–Te···O = 170.86(7)	ChB		
2^c^	Te···I = 4.0509(5)	100.3	C–Te···I = 131.83(10); I–Te···I = 122.464(14)	Type I	IRUQAO	^41^
	Te···Te = 3.4534(5)	83.8	C–Te···Te = 158.54(11)	ChB		
	Te···I = 4.1236(7)	102.1	C–Te···I = 131.77(11); I–Te···I = 123.758(18)	Type I		
	Te···Te = 3.4503(5)	83.8	C–Te···Te = 158.02(11)	ChB		
3	Te···I = 4.3291(5)	107.2	C–Te···I = 151.78(13)	ChB	WUMFAO	^42^
	I···N = 2.772(5)	76.8	C–I···N = 171.37(18)	HaB		
	I···N = 2.818(5)^d^	78.1	C–I···N = 174.6(2)	HaB		
4	Te···I = 3.9995(9)	99.0	P–I···Te = 158.39(2)	HaB	RADRIZ	^43^
5	Te···I = 3.8947(15)	96.4	C–Te···I = 173.0(3)	ChB	POYGIT	^44^
	Te···N = 2.252(9)	76.8	I–Te···N = 169.1(2)	ChB		
6	Te···I = 4.4356(10)	109.8	C–Te···I = 134.7(2); I–Te···I = 80.8(2)	Type I	VOMVEY	^45^
	Te···O = 2.369(6)	66.2	I–Te···O = 169.52(15)	ChB		
7	Te···I = 2.8255(14)	69.9	I–I···Te = 171.15(2)	HaB	FUSWAS	^46^
	Te···I = 3.3449(19)	82.8	I–Te···I = 174.26(3)	ChB		
8	Te···I = 3.7739(14)	93.4	C–Te···I = 164.33(7)	ChB	GEDSEO	^47^
	Te···S = 2.6075(9)	67.6	I–Te···S = 178.576(16)	ChB		
9	Te···I = 3.9132(3)	96.7	C–Te···I = 166.38(4)	ChB	KAJXUS	^48^
	Te···S = 2.4919(5)	64.6	I–Te···S = 177.369(14)	ChB		
10	Te···I = 3.7112(12)	91.9	P···Te···I = 171.83(3)	ChB	CEMMAJ	^49^
	Te···P = 2.4899(13)	61.6	I···Te···P = 171.83(3)	ChB		

a %(d/vdW) = [d­(Te···X)/Σ­(vdW)] × 100, where Σ­(vdW) is the sum of the respective van der Waals radii.

b Abbreviations: NCI, noncovalent interaction; HaB, halogen bond; ChB, chalcogen bond.

c Two independent molecules comprise the crystallographic asymmetric unit.

d Interaction external to the {···TeC_4_N···I}_2_ synthon.

The aggregate in **1** ^40^ is the sole example where a single Te···I interaction is formed between the two independent molecules comprising the crystallographic asymmetric unit, Figure 1. The Te–I···Te angle [170.313 (14)°] is indicative of a HaB. Each molecule also features intramolecular Te···O interactions with the I–Te···O angles, Table 1, indicating that these are ChBs.

**Figure 1:**
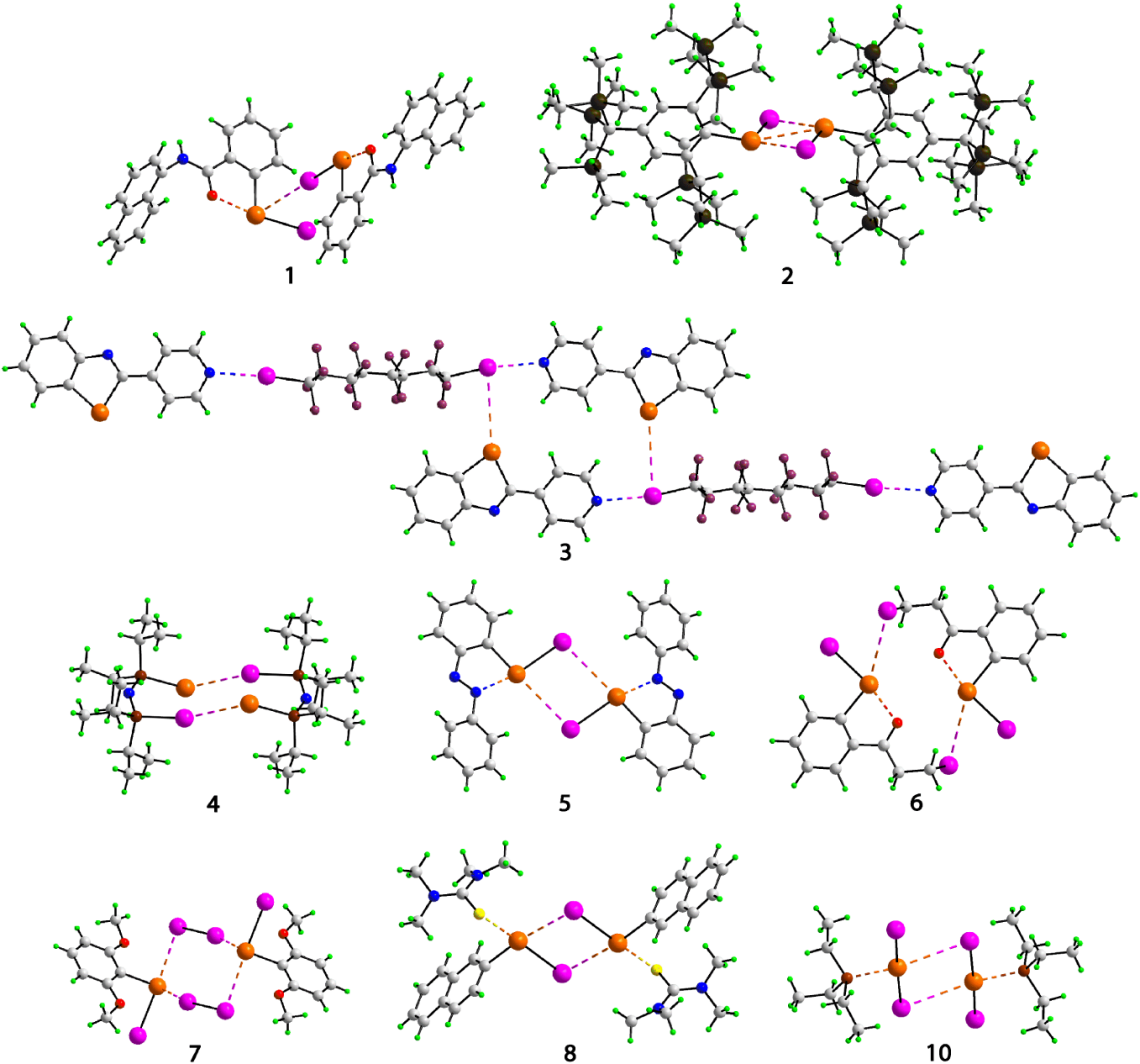
Images of the two-molecule aggregates identified in the crystals of **1**–**8** and **10**. Color code: pink, iodine; orange, tellurium; yellow, sulfur; brown, phosphorus; olive green, silicon; plum, fluorine; red, oxygen; blue, nitrogen; gray, carbon; and bright green, hydrogen. Te···I interactions are highlighted as orange/pink dashed lines: Te···Te, orange; Te···S, orange/yellow; Te···P, orange/brown; Te···O, orange/red; Te···N, orange/blue; I···N, pink/blue.

Two independent Te molecules also comprise the asymmetric unit in **2**, crystallized as a benzene disolvate.^41^ Each independent molecule self-associates about a center of inversion, Figure 1 and SI Figure_ IRUQAO, and features Te···I interactions within a four-membered {···TeI}_2_ synthon, at or just beyond the sum of the van der Waals radii, which are best described as Type I interactions. More significant, based on %(d/vdW) = 83.8, are transannular Te···Te interactions with the C–Te···Te angles suggestive of ChBs, Table 1. The Br congener of **2** is isomorphous with **2,** with details available in SI Table_S1_IRUPUH.^41^ Based on %(*d*/vdW) = values, one Te···Te interaction is as strong as those in **2** while the second Te···Te interaction is significantly weaker but still stronger than the complementary Te···Br interactions. The third aggregate is found in the 2:1 cocrystal, **3**,^42^ and features both Te···I and I···N interactions, Figure 1. One independent 2-(pyridin-4-yl)-1,3-benzotellurazole molecule self-associates about a center of inversion with Te···I interactions; these are ChBs. The iodine atom participating in the Te···I interaction also forms a I···N interaction so a 14-membered {···TeC_4_N···I}_2_ synthon. The second iodine atom of the hexadecafluoro-1,8-di-iodooctane coformer forms an I···N interaction with the second independent 2-(pyridin-4-yl)-1,3-benzotellurazole molecule; both I···N interactions are HaBs. The sole example of a telluride among **1**–**10** is found in the centrosymmetric aggregate, **4**,^43^ Figure 1, where the P–I···Te interaction is consistent with a HaB within a nonplanar, 10-membered {···TePNPI}_2_ synthon. The selenium analog of **4** is known,^43^ SI Figure_S1_RADRAR, and with a %(d/vdW) value (101.9%) greater than that in **4**, the P–I···Se interaction can be considered relatively weaker. By contrast, in the Se/Cl analog of **4**, there is no evidence for ChB or HaB.^70^

The two-molecule, centrosymmetric aggregate in **5** ^44^ is illustrated in Figure 1. The aggregate features a Te···I ChB as well as an intramolecular I–Te···N ChB. A polymorph of **5** is known, as are several congeners. The polymorph of **5**,^71^ SI Figure_S1_POYGIT01, is dominated by intermolecular Te···N ChB within zigzag chains (glide symmetry); the intramolecular Te···N ChB persists. The bromine congener of **5** ^44^ is isomorphous with **5** and while the chlorine congener is not isomorphous with **5**,^44^ the same two-molecule motif persists in both the monoclinic^44^ and triclinic polymorphs;^72^ in the latter, the aggregate involves the two independent molecules comprising the asymmetric unit. In the crystal of the selenium analog of **5**,^73^ molecules assemble into a linear chain with the iodine atom bridging successive selenium atoms. The same motif is observed in the Se/Br analog,^73^ but the two-molecule aggregate of **5** appears in the crystal of the Se/Cl analog^73^ for one of the independent molecules of the asymmetric unit, with the second molecule assembling into a chain; long Se···Se interactions are prominent in the Se/Cl aggregates. However, for the selenium series, reflecting the relatively long Se···X separations, these are Type I contacts in contrast to their tellurium congeners; intramolecular Se···N ChB persists across the selenium series.

The centrosymmetric two-molecule aggregate in **6**,^45^ Figure 1, features the longest Te···I contact distance [4.4356 (10) Å] between molecules among **1**-**10** and accordingly, the Te···I contact may be considered a Type I interaction, which closes a 14-membered {···TeC_5_I}_2_ synthon. The molecule features an intramolecular Te···O interaction which, like the intermolecular Te···N interaction in **5** and its congeners, is a ChB. In **7**,^46^ an I_2_ molecule provides a bridge to link two centrosymmetrically related molecules, Figure 1. One of the Te···I separations is the shortest [2.8255 (14) Å] among **1**-**10** and is considerably shorter than the second Te···I separation [3.3449 (19) Å] in **7**: these are examples of HaB and ChB interactions, respectively. The remaining aggregates to be described each feature external contacts to sulfur (**8** and **9**) or phosphorus (**10**).

The centrosymmetric two-molecule aggregates in the crystals of **8**,^47^ Figure 1, and **9**,^48^ SI Figure_S1_KAJXUS, adopt the same motif, with the tellurium atoms being S-coordinated by tetramethylthiourea and thiourea molecules, respectively. With angles approaching 180°, the C–Te···I interactions within a {···TeI}_2_ synthon are ChBs Table 1. As for the molecules featuring intramolecular Te···N–O interactions, the iodine atom occupies a position approximately trans to the sulfur atoms in **8** and **9**, consistent with ChBs. While there are no congeners for **8**, the bromine and chlorine congeners of **9** have been structurally characterized.^74^ The Te···X interactions for the isomorphous pair, i.e., Te/Br (%(*d*/vdW) = 90.0%) and Te/Cl (90.1%), are marginally stronger.^74^ However, being reported in 1966, it is likely the standard uncertainty values (not available) for these contacts are rather high.^74^ The final aggregate to be described, **10**,^49^ is also located about a center of inversion, Figure 1, and features a central {···TeI}_2_ synthon. The iodine atom forming the intermolecular contact with the tellurium atom occupies a position approximately trans to the phosphorus of the triethylphosphane molecule, and the interaction is classified as a ChB as are the Te···P interactions. The Te···X interactions for Te/Br (%(*d*/vdW) = 90.0%) and Te/Cl (90.1%) congeners are marginally stronger, but the Te/Br (%(*d*/vdW) = 64.1%) and Te/Cl (63.9%) contacts are relatively weaker.^49^

Across the series **1**–**10**, the tellurium atom forms a single Te···I interaction only in **1** (first independent molecule), **3** and **4** whereas in **7**, two Te···I interactions are formed. The tellurium atoms form two contacts in each of **1** (second independent molecule), **2**, **5**–**10** with the second interaction in **1**, **5,** and **6** being intramolecular and those in **2**, **7**–**10** being intermolecular. The Te···I interaction is classified as a ChB in **3**, **5**, and **8**–**10**, a HaB in **1**, **4**, and **7**, and a Type I interaction in **2** and **6**. The intra- and intermolecular Te···O, N, S, and Te interactions are ChBs across the series; the I···N interactions in **3** are HaBs. Thus, it can be concluded that Te preferentially forms ChBs as opposed to I forming HaBs in these experimental structures. With respect to interatomic separations, the Te···I distances, based on van der Waals criteria, are generally long, in contrast to the intramolecular Te···N, O and external Te···S distances, which are usually rather short. In order to gain greater insight into the nature of the NCIs operating in **1**–**10**, DFT calculations were conducted on selected examples.

### 3.2 DFT Calculations

Theoretical calculations were carried out on a representative selection of five systems, namely, **3**, **4**, **5**, **6**, and **8** (refcodes WUMFAO, RADRIZ, POYGIT, VOMVEY, and GEDSEO, respectively), to gain greater insight into the nature of the noncovalent interactions described above. This subset covers the different supramolecular behaviors and interaction types (HaB, ChB, and Type I) observed in the CSD survey. For each compound, the molecular electrostatic potential (MEP) surfaces were computed to investigate the electron-rich and electron-poor regions of the interacting molecules and to rationalize the formation of the aggregates. Subsequently, the interaction energies were evaluated, and the contacts were further characterized using QTAIM, NCIplot, and NBO analyses to evaluate the strength and orbital contributions of the contacts.

The computational analysis of compound **3** commences with the investigation of the MEP surfaces of the individual constituents, namely 2-(pyridin-4-yl)-1,3-benzotellurazole and hexadecafluoro-1,8-diiodooctane (Figure 2a and b, respectively). For the Te-species, the MEP surface reveals two σ-holes at the Te-atom with large positive values (+27.6 and +28.2 kcal/mol), which are likely enhanced by the electronic effects of the adjacent aromatic H-atoms. The region corresponding to the tellurium lone pairs (LPs) is negative (−5.0 kcal/mol), while the absolute MEP minimum is located at the pyridyl-N atom (−35.8 kcal/mol). In the diiodo molecule, the MEP maxima are located at the σ-holes of the iodine atoms (+32.9 kcal/mol), and the minima are found at the fluorine atoms (−6.9 kcal/mol). Notably, the negative electron-rich belt usually found around iodine is very weak in this perfluorinated compound (−0.6 kcal/mol), suggesting a very poor electron donor ability, which aligns with the long Te···I distance observed experimentally (see Table 1).

**Figure 2:**
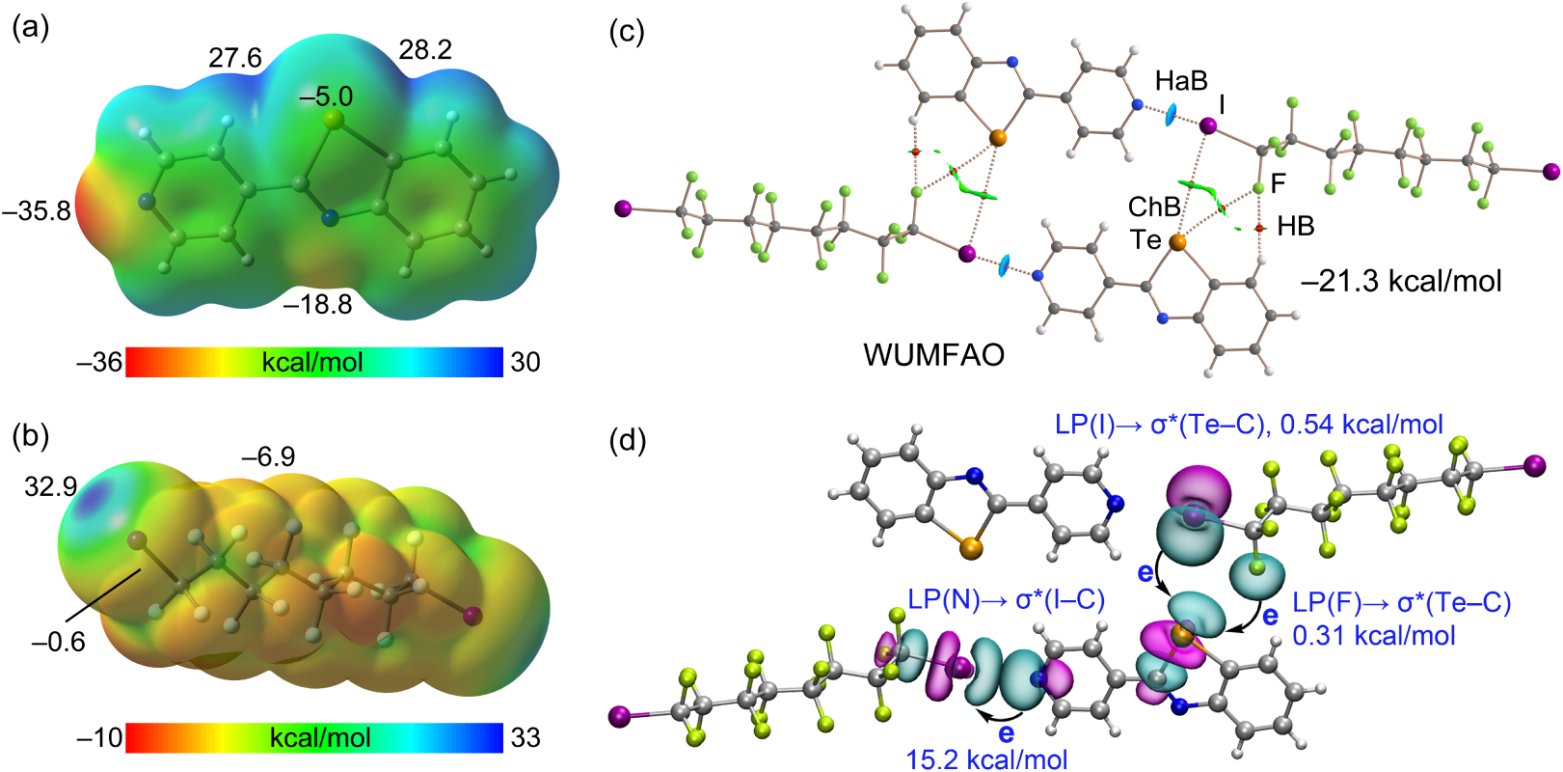
(a,b) MEP surfaces (0.001 au) of 2-(pyridin-4-yl)-1,3-benzotellurazole (a) and hexadecafluoro-1,8-diiodooctane (b). The energies at selected points of the surfaces are given in kcal/mol. (c) QTAIM/NCIplot analysis of the self-assembled tetramer of **3** (WUMFAO). The bond critical points (BCPs) are represented by red spheres, and bond paths by dashed lines. The NCIplot isosurface setting is 0.5, and the color scale is – 0.04 < *ρ* < 0.04 au. (d) NBO analysis of the tetramer of **3** indicating the relevant donor–acceptor orbital interactions and their associated second-order stabilization energies, E^(2)^, in kcal/mol. HB = hydrogen bond.

Figure 2c displays the QTAIM/NCIplot analysis of the tetrameric assembly previously described in Figure 1. The QTAIM analysis confirms the existence of the I···N HaB and the Te···I ChB detected geometrically. Each contact is characterized by a bond critical point (BCP, small red spheres) and a bond path interconnecting the nuclei of the interacting atoms. The interactions are further visualized by reduced density gradient (RDG) isosurfaces. The dark blue color for I···N HaB indicates a strong attractive interaction, whereas the green region corresponding to the Te···I ChB is indicative of a much weaker interaction. Furthermore, the QTAIM/NCIplot analysis discloses the existence of additional contacts that contribute to the stabilization of the assembly. These include a C–H···F hydrogen bond (HB) connecting an aromatic C–H of the benzotellurazole moiety to a fluorine atom of the diiodo-coformer, and a Te···F contact. The latter suggests the formation of a bifurcated Te···I–F chalcogen bond. The computed formation energy of this complete assembly is large (−21.3 kcal/mol), a consequence of this intricate combination of interactions, confirming its importance in the solid state of **3**.

Finally, the NBO analysis (Figure 2d) confirms the charge-transfer nature of the main interactions. For clarity, the HaB is represented in one part of the assembly and the ChB in the opposite part, although both types exist simultaneously. The analysis confirms the LP → σ* orbital overlap common to σ-hole interactions. The second-order perturbation energies (E^(2)^) demonstrate that the I···N HaB is significantly stronger (E^(2)^ = 15.2 kcal/mol) than the ChB contacts. The combined contribution of the bifurcated Te···I and Te···F ChBs is only 0.85 kcal/mol, which is almost negligible compared to that of HaB. This energetic difference is fully consistent with the geometrical parameters, contrasting the short I···N distance with the very long Te···I distance listed in Table 1.

The analysis of the dimer of **4** (RADRIZ) is summarized in Figure 3. The MEP surface of the monomer (N-(diisopropyl­(telluro)­phosphoranyl)-P,P-diisopropylphosphinimidic iodide) is shown in Figure 3a. Notably, the formal Te = P double bond results in a large negative MEP value (−36.4 kcal/mol) at the tellurium atom, which represents the most nucleophilic site of the molecule. Consequently, no positive σ-hole is observed at the Te-atom, though the MEP distribution is anisotropic. In contrast, the iodine atom presents a modest σ-hole of +11.3 kcal/mol. Interestingly, the potential at the negative belt of the iodine atom is negligible (∼0 kcal/mol), while the most positive regions of the surface are located at the aliphatic H atoms (+25.1 kcal/mol). The negligible potential at the iodine belt facilitates the formation of the Type I I···I contact [4.1162 (9) Å] observed within the self-assembled dimer, as the electrostatic repulsion between the belts is minimized, allowing dispersion forces to dominate.

**Figure 3:**
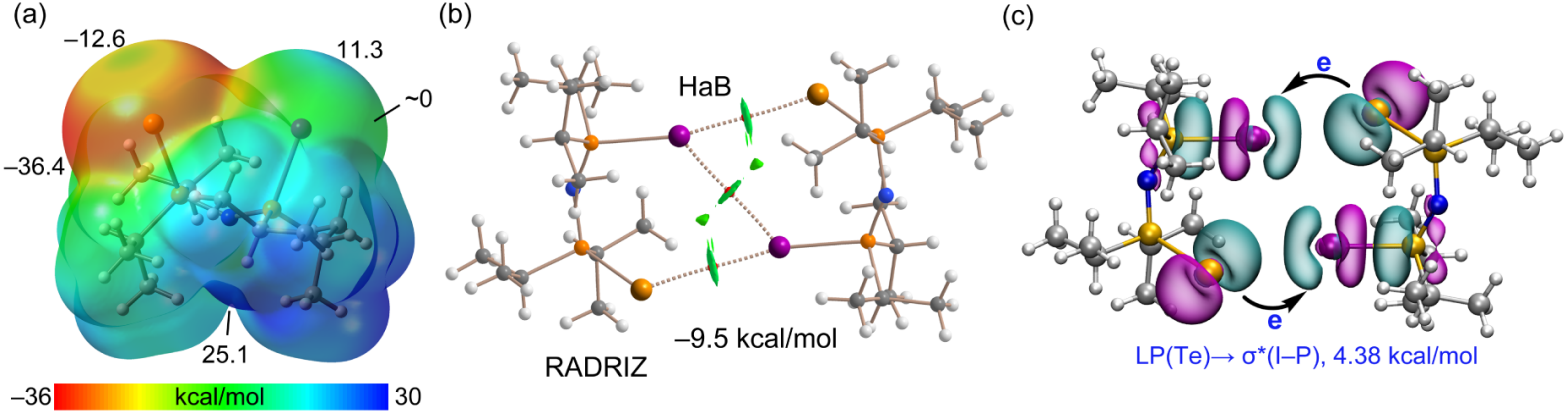
(a) MEP surface (0.001 au) of the monomer of **4** (RADRIZ), with energies at selected points given in kcal/mol. (b) QTAIM/NCIplot analysis of the centrosymmetric dimer of **4**. The BCPs are represented by red spheres, and bond paths are represented by dashed lines. (c) NBO analysis of the dimer of **4,** showing the relevant LP­(Te) → σ*­(I–P) orbital interactions and the associated second-order stabilization energy, E^(2)^, in kcal/mol.

The QTAIM/NCIplot analysis (Figure 3b) confirms the existence of both the Te···I contacts and the Type I I···I interaction. Each is characterized by a bond critical point (BCP) and a bond path connecting the interacting atoms. The reduced density gradient (RDG) isosurfaces appear green, indicating the weak, noncovalent nature of these interactions. The dimerization energy is moderate (−9.5 kcal/mol), which is reasonable considering the formation of two HaBs and the additional dispersive I···I contacts.

The NBO analysis (Figure 3c) provides further insight into the nature of the Te···I interaction. It confirms a HaB mechanism where an LP orbital of the nucleophilic Te-atom donates charge to the σ* orbital of the P–I bond. The E^(2)^ value for this LP­(Te) → σ*­(P–I) interaction is substantial (4.38 kcal/mol). However, this value is significantly smaller than the HaB energy calculated for compound **3** (15.2 kcal/mol), a reduction attributed to the longer Te···I distance and the weaker σ-hole donor ability of the iodine atom in **4**.

The computational results for compound **5** (POYGIT) are summarized in Figure 4. This system is interesting because the iodido (2-phenylazophenyl-C,N) tellurium molecule can exist in two resonance forms, namely, neutral and close-contact ion-pair (Figure 4a). The experimental intramolecular Te–N distance (2.252 (9) Å, see Table 1) is very close to the sum of the covalent radii of Te and N (ΣR_cov_ = 2.09 Å),^75^ suggesting a significant degree of covalency and the dominance of the close-contact ion-pair form in the solid state. This hypothesis is strongly supported by the MEP analysis (Figure 4b), which shows the absolute minimum located at the iodine atom (−23.8 kcal/mol). Consequently, the region at the extension of the Te–I bond (the σ-hole) is also negative (−11.9 kcal/mol). Conversely, the MEP maximum is located at the aromatic H-atoms (+22.0 kcal/mol), and a large positive potential (+20.1 kcal/mol) is observed at the tellurium σ-hole opposite the aromatic C-atom. The covalent nature of the intramolecular Te–N bond is further corroborated by the QTAIM analysis, which reveals a large charge density at the bond critical point (ρ = 0.0808 au) and a negative total energy density (H = −0.0161 au). Additionally, the NCIplot analysis shows an absence of RDG isosurfaces between the Te and N atoms, which is typical for covalent bonds. Regarding the intermolecular interactions, the QTAIM analysis of the centrosymmetric dimer (Figure 4c) reveals two symmetric Te···I ChBs characterized by the corresponding BCPs and bond paths. The reduced density gradient isosurfaces are disk-shaped and green, characteristic of noncovalent interactions. The analysis also discloses the existence of secondary C–H···I HBs, which are electrostatically favored due to the negative potential at the iodine atom and the positive character of the aromatic protons. These auxiliary interactions likely contribute to a moderately strong dimerization energy of −8.3 kcal/mol. Finally, the NBO analysis (Figure 4d) confirms the σ-hole ChB nature of the Te···I interaction, showing a clear LP­(I) → σ*­(Te–C) charge donation with an associated second-order stabilization energy of 3.62 kcal/mol for each contact.

**Figure 4:**
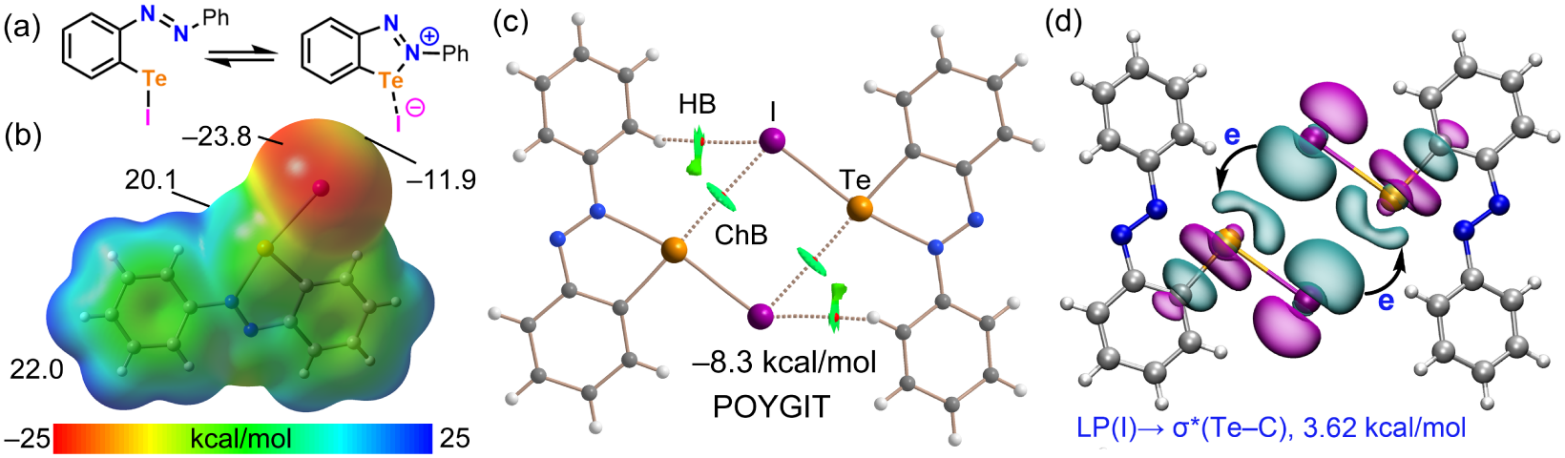
(a) Neutral and ion-pair forms of **5**. (b) MEP surface (0.001 au) of **5,** with energies at selected points given in kcal/mol. (c) QTAIM/NCIplot analysis of the centrosymmetric dimer of **5**. The BCPs are represented by red spheres, and bond paths by dashed lines. (d) NBO analysis of the dimer of **5,** showing the relevant LP­(I) → σ*­(Te–C) orbital interactions and the associated second-order stabilization energy, E^(2)^, in kcal/mol.

The analysis of **6** (VOMVEY) is presented in Figures 5 and 6. Similar to the previous case, the o-(3-iodopropionyl)­phenyl-iodido-tellurium molecule can be described by an equilibrium between neutral and close-contact ion-pair forms (Figure 5a). The experimental intramolecular Te–O distance [2.369 (6) Å] is much closer to the sum of the covalent radii (2.04 Å) than to the sum of the van der Waals radii (3.58 Å), supporting a strong contribution from the close-contact ion-pair structure. This interpretation is further corroborated by the QTAIM analysis of the intramolecular Te–O bond, which reveals a bond critical point with ρ = 0.0545 au and a negative total energy density (H = −0.0122 au), indicative of significant covalent character. This interpretation is further corroborated by the MEP surface analysis (Figure 5b). The surface shows a deep minimum at the negative belt of the iodine atom bonded to tellurium (−19.5 kcal/mol), which also exhibits a negative σ-hole. A significant negative potential is also observed at the oxygen atom (−16.3 kcal/mol). Conversely, the global MEP maximum (+33.3 kcal/mol) is located at the hydrogen atoms of the iodoethyl chain adjacent to the positive carbonyl carbon, consistent with charge separation in the ion-pair form. The σ-hole at the Te atom (opposite to the Te–C bond) is positive but relatively small (+8.5 kcal/mol), while the σ-hole at the aliphatic iodine atom is significantly more positive (+16.3 kcal/mol). Two different centrosymmetric dimers observed in the solid state were analyzed. Figure 5c shows the QTAIM/NCIplot representation of the first dimer, which is stabilized by two intermolecular Te···O interactions. The dimerization energy is modest (−4.3 kcal/mol), in line with the small green RDG isosurfaces and the weak positive potential at the tellurium σ-hole. The second dimer (Figure 5d) features I···I HaB interactions between the nucleophilic iodine bonded to the Te-atom and the electrophilic aliphatic iodine. These contacts are characterized by BCPs, bond paths connecting the iodine atoms, and disk-shaped RDG isosurfaces. The dimerization energy for this assembly is −6.4 kcal/mol, indicating that HaBs are stronger than ChBs in the first dimer. This energetic hierarchy agrees well with the MEP analysis, which showed that the aliphatic iodine σ-hole is more positive than the tellurium σ-hole and that the iodine bonded to tellurium is more negative than the oxygen atom.

**Figure 5:**
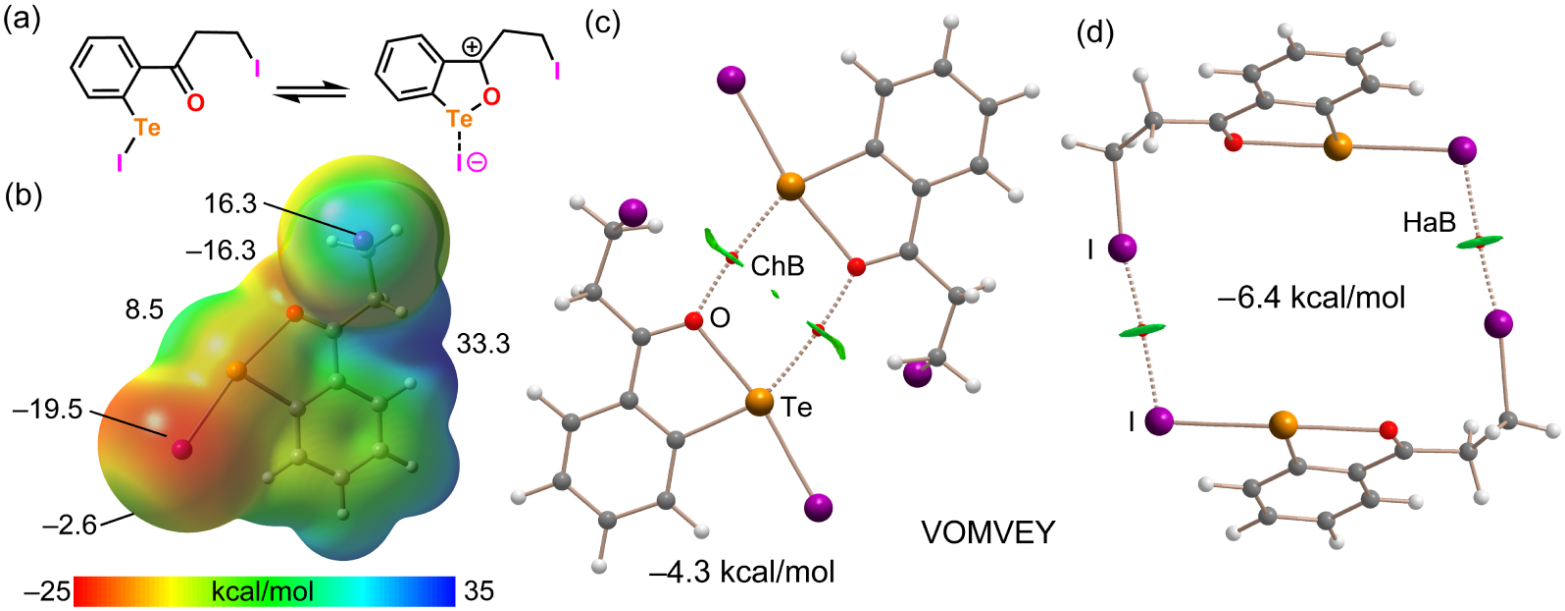
(a) Neutral and ion-pair forms of **6**. (b) MEP surface (0.001 au) of **6** with energies at selected points given in kcal/mol. (c) QTAIM/NCIplot analysis of the centrosymmetric Te···O bonded dimer. (d) QTAIM/NCIplot analysis of the centrosymmetric I···I bonded dimer. The BCPs are represented by red spheres, and bond paths by dashed lines.

**Figure 6:**
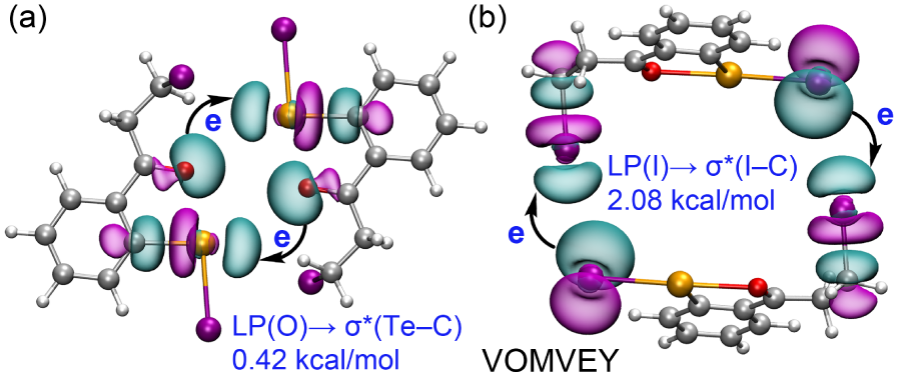
NBO analysis of the Te···O bonded dimer (a) and the I···I bonded dimer (b) of **6**, showing the relevant donor–acceptor orbital interactions and their associated second-order stabilization energies, E^(2)^, in kcal/mol.

The NBO analysis for both dimers is depicted in Figure 6. For the ChB dimer (Figure 6a), the charge transfer from the lone pair of the oxygen atom to the antibonding σ*­(Te–C) orbital is modest (E^(2)^ = 0.42 kcal/mol), likely due to the long interaction distance and poor directionality (see Table 1). In contrast, the NBO analysis of the HaB dimer (Figure 6b) confirms a more significant electron donation from an LP orbital of the iodine bonded to tellurium to the σ*­(I–C) orbital of the aliphatic moiety. The stabilization energy is 2.08 kcal/mol for each I···I contact. These NBO results align perfectly with the dimerization energies and MEP analysis, confirming the predominant role of HaB in the supramolecular organization of the VOMVEY structure.

The computational study concludes with an analysis of compound **8** (GEDSEO), illustrated in Figure 7. This system presents an interesting case, as it can be viewed either as a 1:1 cocrystal of N,N,N’,N’-tetramethylthiourea and iodido-(2-naphthyl)­tellurium­(II), or as a close-contact ion-pair (Figure 7a). The experimental Te–S distance is 2.6075 (9) Å, which is remarkably close to the sum of the covalent radii (ΣR_cov_ = 2.43 Å). In fact, the original structural determination described the tellurium atom as tricoordinated. Consequently, for the purpose of this analysis, the iodido-(N,N,N’,N’-tetramethylthiourea-S)-(2-naphthyl)­tellurium­(II) unit has been treated as a discrete monomeric species. To understand the formation of this monomer, the MEP surface of the iodido-(2-naphthyl)­tellurium­(II) precursor was computed (Figure 7b). It reveals a deep σ-hole at the tellurium atom opposite the Te–I bond (+30.1 kcal/mol), which is responsible for the formation of the strong and predominantly covalent Te–S bond. Upon interaction with the thiourea ligand, the electronic distribution changes significantly (Figure 7c). The iodine atom becomes highly negative, indicative of significant charge transfer from the sulfur atom to iodine. Consequently, the positive σ-hole opposite the Te–S bond disappears. However, the second σ-hole at the Te-atom (opposite the naphthyl C-atom) remains positive (+3.8 kcal/mol), although its value is reduced compared to the precursor (+15.1 kcal/mol). The QTAIM/NCIplot analysis of the self-assembled dimer observed in the crystal structure is depicted in Figure 7d. The analysis confirms the existence of two symmetric Te···I ChBs connecting the monomers. The interaction energy is considerable (−7.8 kcal/mol), which is somewhat surprising, given the small magnitude of the remaining σ-hole at the tellurium atom (+3.8 kcal/mol). This binding strength is likely attributed to the excellent directionality of the interaction and the strongly negative MEP of the iodine atom, which effectively compensates for the weaker electrophilicity of the tellurium σ-hole. Finally, the NBO analysis (Figure 7e) reveals a considerable electron donation from an LP­(I) to the antibonding σ*­(Te–C) orbital of the partner molecule. The E^(2)^ is 5.3 kcal/mol for each Te···I contact. This result not only confirms the σ-hole nature of the ChB but also highlights the significant orbital contribution to the total dimerization energy.

**Figure 7:**
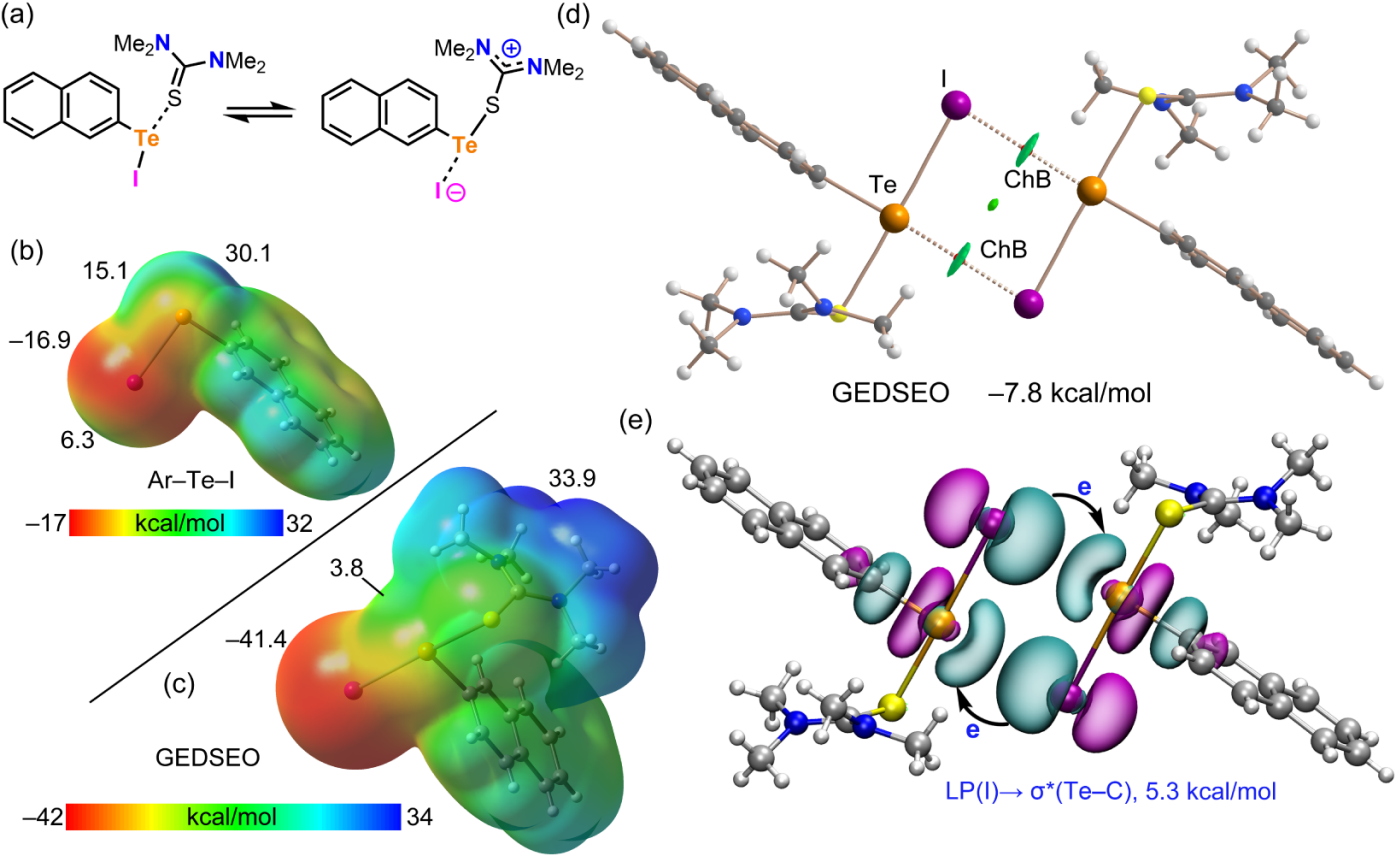
(a) Equilibrium between the neutral cocrystal and ion-pair forms of **8**. (b) MEP surface (0.001 au) of the iodido-(2-naphthyl)­tellurium­(II) precursor. (c) MEP surface of the monomer of **8**. Energies at selected points are given in kcal/mol. (d) QTAIM/NCIplot analysis of the centrosymmetric dimer of **8**. The BCPs are represented by red spheres, and bond paths by dashed lines. (e) NBO analysis of the dimer of **8,** showing the relevant LP­(I) → σ*­(Te–C) orbital interactions and the associated second-order stabilization energy in kcal/mol.

## 4 Conclusions

The present survey of the CSD has identified ten examples of two-molecule aggregates, **1**–**10**, featuring Te···I interactions between the constituent molecules. The structural analysis reveals a rich diversity of supramolecular behaviors, where the Te···I contacts can be classified as halogen bonds (HaB), chalcogen bonds (ChB), or Type I interactions depending on the geometric disposition of the interacting atoms. A preference for Te···I ChB formation is observed when the tellurium­(II) atom is involved, as seen in **3**, **5**, **8**, **9**, and **10**. In contrast, HaB interactions are prominent in **1**, **4**, and **7**, while Type I contacts are restricted to **2** and **6**. When tellurium­(II) forms additional contacts with N, O, S, and Te, internal or external to the molecule, the interaction is classified as a ChB, revealing a propensity for tellurium­(II) to engage in ChB formation in the surveyed experimental structures. The detailed comparison with lighter congeners (S, Se and F, Cl, and Br) highlights the propensity of tellurium and iodine to engage in these specific secondary-bonding interactions, leading to isostructurality in a limited number of subsets of analogous compounds. The DFT calculations performed on selected systems (**3**, **4**, **5**, **6**, and **8**) have provided crucial insights into the electronic nature of these assemblies. The results confirm that the interplay between intermolecular Te···I interactions and intramolecular or external coordination significantly modulates the σ-hole donor and nucleophilic acceptor properties of the interacting sites. Notably, the computations support the description of compounds **5**, **6**, and **8** as close-contact ion pairs, a feature that enhances the electrostatic component of the binding. The energetic analysis reveals that while some aggregates are stabilized by robust HaBs (e.g., in **3**), others rely on the cooperative effect of weaker ChBs and auxiliary interactions such as hydrogen bonds or Type I contacts. The QTAIM and NBO analyses consistently validate the noncovalent nature of these interactions, underscoring the dominance of orbital charge transfer effects (LP → σ*) in the stabilization of both HaB and ChB motifs. Overall, this study demonstrates that the supramolecular organization of organotellurium iodides is governed by a delicate balance of competing forces. The ability to tune these interactions through chemical modification offers a valuable tool for crystal engineering, allowing for the predictable design of materials with specific structural motifs.

## Supplementary Material



## Notes

Descriptions, geometric parameters, and relevant diagrams for additional interactions involving Te and/or I, and for congeners of **1**–**10** (PDF)
