# Supplementary material for: Interplay between Inter- and Intramolecular Halogen-/Chalcogen-Bonding in Two-Molecule Aggregates Featuring TeII···I Secondary-Bonding Interactions and in Their Congeners
Source: J Phys Chem A. 2026 May 5;130(20):3868–78. doi: 10.1021/acs.jpca.6c00421 (PMC13200182; doi:10.1021/acs.jpca.6c00421)
Supplement: Supplementary file 1 [file jp6c00421_si_001.pdf]

Interplay between Inter- and Intramolecular Halogen-/Chalcogen-Bonding in Two-Molecule Aggregates Featuring $Te^{II}\cdots I$ Secondary-Bonding Interactions and in their Congeners†

Rosa M. Gomila, Antonio Frontera* and Edward R. T. Tiekink*

Department of Chemistry, Universitat de les Illes Balears, Crta de Valldemossa km 7.5, 07122 Palma de Mallorca, Spain.

E-mail: rosa.gomila@uib.es (RMG); toni.frontera@uib.es (AF); edward.tiekink@uib.es (ERTT)

Table S1. Diagrams and selected geometric data for the six crystals, **1–10**, identified as forming two-molecule aggregates incorporating Te···I interactions and, where relevant, equivalent data and analysis of congeners of these crystals.

Color code: pink, iodine; orange, tellurium; yellow, selenium, light blue; bromine, dark yellow; chlorine, cyan; sulfur; brown, phosphorus; olive green, silicon; plum, fluorine; red, oxygen; blue, nitrogen; gray, carbon; bright-green, hydrogen. Te···I interactions are highlighted as orange/pink dashed lines, Te···Te, orange; Te···S, orange/yellow; Te···P, orange/brown; Te···O, orange/red; Te···N, orange/blue; I···N, pink/blue.

1_HIRRIN iodo-{2-[(naphthalen-1-yl)carbamoyl]phenyl}-tellurium

Jain, S.; Batabyal, M.; Thorat, R. A.; Choudhary, P.; Jha, R. K.; Kumar, S. 2-Benzamide Tellurenyl Iodides: Synthesis and Their Catalytic Role in CO_2 Mitigation. *Chem. – A Eur. J.* **2023**, *29*, e202301502.

Triclinic, $P\bar{1}$, a = 8.4162(6), b = 12.9855(9), c = 15.5015(11) Å, α = 105.873(2), β = 90.816(2), γ = 98.163(2)°, V = 1610.5(2) Å^3, Z' = 2, T = 140 K

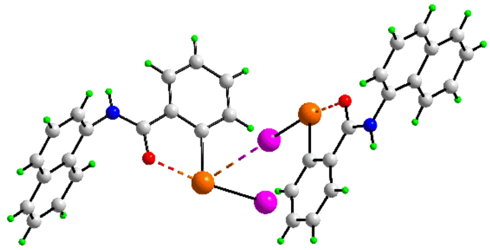

Te···I = 3.8454(4) Å; Te–I···Te = 170.313(14)°

Te···O = 2.384(3) Å; I–Te···O = 170.71(7)°

Te···O = 2.382(3) Å; I–Te···O = 170.86(7)°

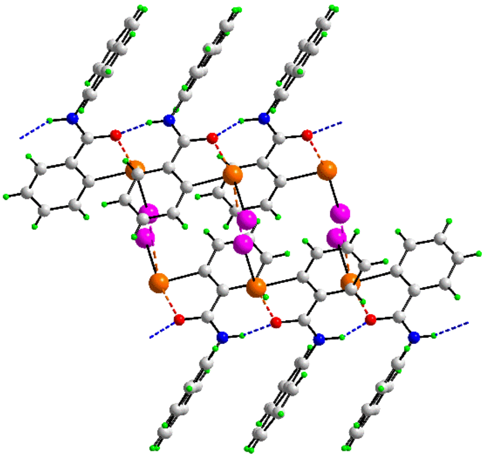

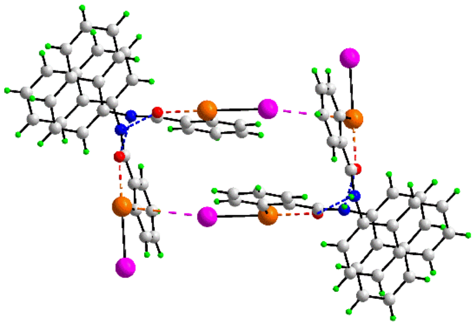

{Two independent molecules. Two-molecule aggregate with a single Te···I interaction between the independent molecules. Each molecule forms an intramolecular Te···O interaction within a non-symmetric 5-membered {···TeOC$_3$} synthon. The aggregates assemble into chains with a tubular topology and feature amide-N–H···O(amide) hydrogen bonds between them [N–H = 2.21 Å and angle at H = 141°; 2.15 Å & 135°] shown as blue dashed lines}

NO CONGENERS.

2_IRUQAO iodido-(2,6-bis(bis(trimethylsilyl)methyl)-4-(tris(trimethylsilyl)methyl)phenyl)-tellurium benzene mono-solvate

Sasamori, T.; Sugamata, K.; Tokitoh, N. Halogenation Reactions of a Ditelluride Having Bulky Aryl Groups Leading to the Formation of Organotellurium Halides. *Heteroatom Chem*. **2011**, *22*, 405–411.

Triclinic, $P\bar{1}$, a = 12.3795(4), b = 18.9885(5), c = 21.6496(5) Å, α = 100.0553(11), β = 94.0338(12), γ = 103.869(3)°, V = 4831.25(24) Å^3, Z' = 2, T = 103 K

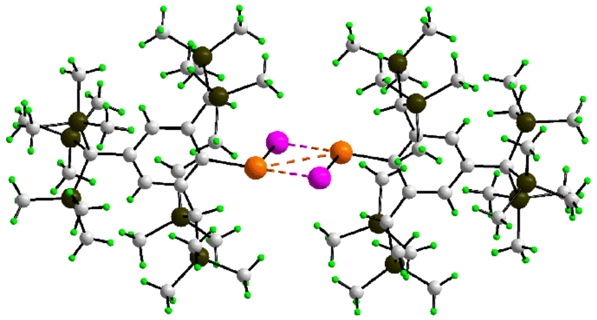

Te···I = 4.0509(5) Å; %(d/vdW) = 100.3; C–Te···I = 131.83(10); I–Te···I = 122.464(14)°

Te···Te = 3.4534(5) Å; %(d/vdW) = 83.8; C–Te···Te = 158.54(11)

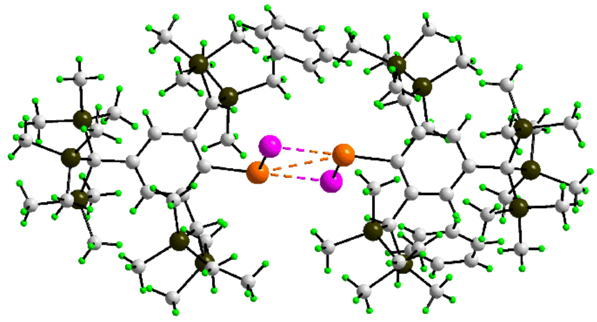

Te···I = 4.1236(7) Å; %(d/vdW) = 102.1; C–Te···I = 131.77(11); I–Te···I = 123.758(18)°

Te···Te = 3.4503(5) Å; %(d/vdW) = 83.8; C–Te···Te = 158.02(11)° {second independent molecule} {Two independent molecules. Each forms a two-molecule aggregate about a center of inversion with two Te···I interactions between them. No further close intermolecular contacts involving either of the Te and I atoms}

CONGENER:

IRUPUH bromido-(2,6-bis(bis(trimethylsilyl)methyl)-4-(tris(trimethylsilyl)methyl)phenyl)-tellurium benzene mono-solvate

Sasamori, T.; Sugamata, K.; Tokitoh, N. Halogenation Reactions of a Ditelluride Having Bulky Aryl Groups Leading to the Formation of Organotellurium Halides. *Heteroatom Chem*. **2011**, *22*, 405–411.

Triclinic, $P\bar{1}$, a = 12.3761(2), b = 18.9663(2), c = 21.6485(2) Å, α = 99.9955(5), β = 94.1515(5), γ = 103.9852(11)°, V = 4821.17(11) Å^3, Z' = 2, T = 103 K

Te···Br = 4.0479(7) Å; C–Te···Br = 131.84(16); Br–Te···Br = 122.575(18)°; Te···Te = 3.4503(5) Å; C–Te···Te = 3.4503(5) Å; C–Te···Te = 157.98(15)°

Te···Br = 4.1062(9) Å; C–Te···Br = 131.77(11); I–Te···I = 123.758(18)°; Te···Te = 3.4533(5) Å; C–Te···Te = 3.4533(5) Å; C–Te···Te = 158.52(15)° {second independent molecule}

{Isomorphous with **IRUQAO**. %(d/vdW) = 103.5 & 105.0 for Te···Br and 83.8 & 99.7 for Te···Te. No further close intermolecular contacts involving either of the Te and Br atoms}

3_WUMFAO bis(2-(pyridin-4-yl)-1,3-benzotellurazole) 1,1,2,2,3,3,4,4,5,5,6,6,7,7,8,8-hexadecafluoro-1,8-di-iodooctane

Biot, N.; Bonifazi, D. Concurring Chalcogen-and Halogen-Bonding Interactions in Supramolecular Polymers for Crystal Engineering Applications. *Chem. – A Eur. J.* 2020, *26*, 2904–2913.

Triclinic, $P\bar{1}$, a = 7.4276(4), b = 14.6567(8), c = 17.4866(11) Å, α = 81.685(5), β = 85.132(5), γ = 79.882(5)°, V = 1850.90(19) Å^3, Z' = 1, T = 150 K

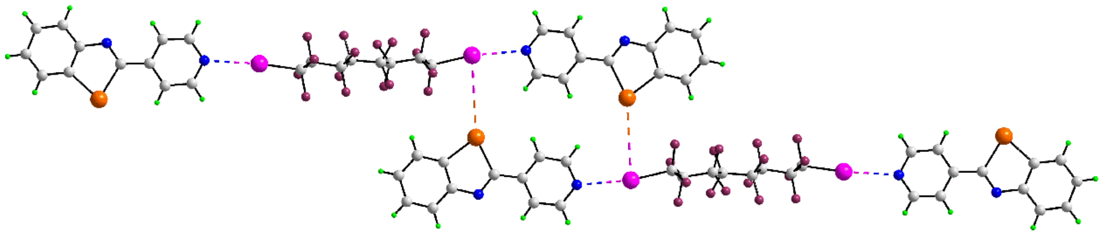

Te···I = 4.3291(5) Å; C–Te···I = 151.78(13)°

I···N = 2.772(5) Å; C–I···N = 171.37(18)°

I···N = 2.818(5) Å; C–I···N = 174.6(2)° {external to the {···TeC_4N···I}$_2$ synthon}

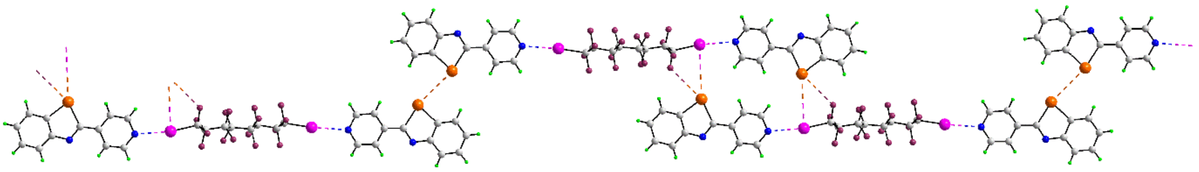

{Two independent 2-(pyridin-4-yl)-1,3-benzotellurazole molecules one of which is disposed about a center of inversion to form a two-molecule aggregate; the second 2-(pyridin-4-yl)-1,3-benzotellurazole molecule does not participate in Te···I interactions. The two molecule aggregates are linked by I···N halogen bonds involving the iodine atom forming the contact to tellurium. The Te···I and I···N interactions occur within a 14-membered {···TeC_4N···I}$_2$ synthon. The six molecule aggregates are connected into a chain via Te···Te type I interactions over a center of inversion [Te···Te = 3.8823(6) Å; 157.44(16)°]. Additional Te···F contacts (shown as orange/plum dashed lines) are noted at a separation longer than the sum of the van der Waals radii (3.53 Å): Te···F = 3.542(4) Å; 153.06(15)°. These involve the tellurium atom participating in the Te···I interactions}

NO CONGENERS.

4_RADRIZ N-(diisopropyl(telluro)phosphoranyl)-P,P-diisopropylphosphinimidic iodide

Ritch, J. S.; Robertson, S. D.; Risto, M.; Chivers, T. Synthesis, Multinuclear NMR Spectra, and X-ray Structures of ^{t}Bu$_2$PNP(I)^{t}Bu$_2$ and EPR$_2$NP(I)R$_2$ (E = Se, Te; R = ^{i}Pr, ^{t}Bu). *Inorg. Chem.* **2010**, *49*, 4681–4686.

Monoclinic, $P2_1/n$, a = 7.5215(15), b = 17.514(4), c = 14.323(3) Å, β = 101.00(3)°, V = 1852.1(7) Å^3, Z' = 1, T = 173 K

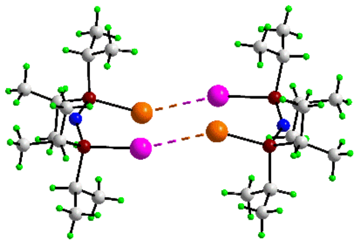

Te···I = 3.9995(9) Å; %(d/vdW) = 99.0; P–I···Te = 158.39(2)°

{Centrosymmetric, 10-membered {···TePNPI}$_2$ synthon with an extended, flattened chair conformation; no close intermolecular contacts involving the Te and I atoms}

CONGENERS:

RADRAR N-(diisopropylphosphoroselenoyl)-P,P-diisopropyl-phosphinimidic iodide

Ritch, J. S.; Robertson, S. D.; Risto, M.; Chivers, T. Synthesis, Multinuclear NMR Spectra, and X-ray Structures of $^{t}Bu_2PNP(I)^{t}Bu_2$ and $EPR_2NP(I)R_2$ (E = Se, Te; R = ^{i}Pr, ^{t}Bu). *Inorg. Chem.* **2010**, *49*, 4681–4686.

Monoclinic, $P2_1/n$, a = 7.4672(3), b = 17.2825(9), c = 14.2260(5) Å, β = 102.665(3)°, V = 1791.22(14) Å^3, Z' = 1, T = 173 K

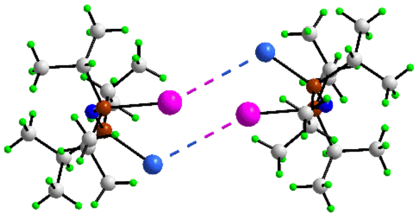

d(Se···I) = 3.9520(6) Å; %(d/vdW) = 101.9; P–I···Se = 157.56(3)°

{Se, sky blue; Se···I interaction, pink/light blue dashed lines. Isomorphous with **RADRIZ**. No close intermolecular contacts involving the Se and I atoms}

YETWEA N-(chlorido(diisopropyl)phosphine)-N-(diisopropylphosphine selenoxide)imine

Konu, J.; Chivers, T.; Tuononen, H. M. Synthesis, Spectroscopic, and Structural Investigation of the Cyclic $[N(PR_2E)_2]^+$ Cations (E = Se, Te; R = iPr, Ph): The Effect of Anion and R-group Exchange, *Inorg. Chem.* **2006**, *45*, 10678–10687.

Orthorhombic, $P2_12_12_1$, a = 7.0377(14), b = 13.483(3), c = 18.421(4) Å, V = 1748.0(6) Å^3, Z' = 1, T = 173 K

{No intermolecular Se···Cl < 5.0 Å; the Se atom is located in a hydrogen-rich pocket with the closest Se···H contact being 3.07 Å; the same is true for the Cl atom, with the closest Cl···H contact being 2.83 Å}

5_POYGIT (2-phenylazophenyl)-iodo-tellurium(II)

Majeed, Z.; McWhinnie, W. R.; Hamor, T. A. Observations on the Synthesis and Chemistry of a Complete Series of Phenylazophenyl (C, N′) Tellurium (II) Halides (Fluoride, Chloride, Bromide and Iodide). *J. Organom. Chem.* **1997**, *549*, 257–262.

Triclinic, $P\bar{1}$, a = 8.011(2), b = 11.228(2), c = 7.677(2) Å, α = 105.81(3), β = 91.60(2), γ = 73.85(3)°, V = 637.3(3) Å, Z' = 1, T = r.t.

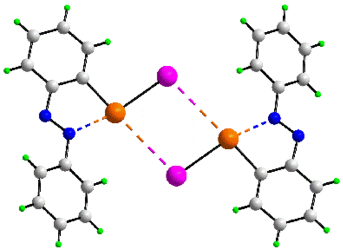

Te···I = 3.8947(15) Å; %(d/vdW) = 96.4%; C–Te···I = 173.0(3)°

Te···N = 2.252(9) Å; %(d/vdW) = 62.4%; I–Te···N = 169.1(2)°

{Centrosymmetric {···TeI}$_2$ synthon. Intramolecular Te···N interaction [2.252(9) Å]}

POLYMORPH:

POYGIT01 (2-phenylazophenyl)-iodido-tellurium(II)

Srivastava, K.; Shah, P.; Singh, H. B.; Butcher, R. J. Isolation and Structural Characterization of Some Aryltellurium Halides and Their Hydrolyzed Products Stabilized by an Intramolecular Te···N Interaction, *Organometallics* **2011**, *30*, 534–546.

Monoclinic, $P2_1/c$, a = 9.2451(6), b = 12.6258(10), c = 10.7974(8) Å, β = 99.882(4)°, V = 1241.65(16) Å, Z' = 1, T = r.t.

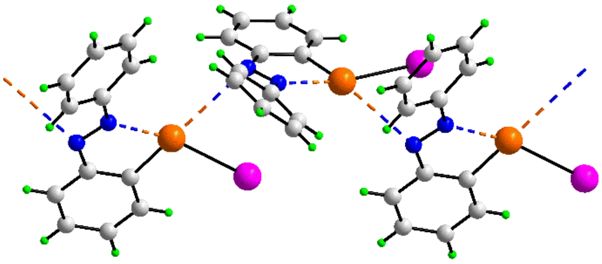

Te···N = 3.3451(15) Å; %(d/vdW) = 92.7%; C–Te···N = 160.22(5)°

Te···N = 2.2103(16) Å; %(d/vdW) = 61.2%; I–Te···N = 168.17(4)° {intramolecular}

{A zigzag chain (glide symmetry) features close Te···N interactions, an intramolecular Te···N interaction is also evident. The closest Te···I contact is > 4.5 Å and there is no I···I contact < 5.0 Å}

CONGENERS:

POYGEP (2-phenylazophenyl-C,N)-bromido-tellurium(II)

Z. Majeed, W. R. McWhinnie and T. A. Hamor, Observations on the Synthesis and Chemistry of a Complete Series of Phenylazophenyl (C, N′) Tellurium (II) Halides (Fluoride, Chloride, Bromide and Iodide), *J. Organomet. Chem.* **1997**, *549*, 257–262.

Triclinic, $P\bar{1}$, a = 7.897(2), b = 11.022(2), c = 7.537(2) Å, α = 105.08(2), β = 91.04(1), γ = 74.46(2)°, V = 609.3(3) Å, Z' = 1, T = r.t.

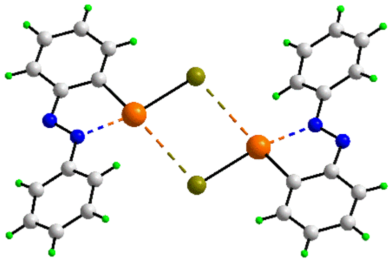

Te···Br = 3.7965(19) Å; %(d/vdW) = 97.1%; C–Te···Br = 174.0(2)°

Te···N = 2.218(9) Å; %(d/vdW) = 61.4%; Br–Te···N = 168.0(2)°

{Isomorphous with **POYGIT**. Centrosymmetric $\{\cdots TeBr\}_2$ synthon incorporating Te···Br interactions shown as orange/dark yellow dashed lines. Intramolecular Te···N interaction [2.218(9) Å]. Closest intermolecular interactions: Te···Te = 4.71 Å; Te···N < 5.0 Å; Br···Br = 4.59 Å; Br···N = 4.54 Å}

PAZPTE01 2-phenylazophenyl-chlorido-tellurium(II) – monoclinic polymorph

Z. Majeed, W. R. McWhinnie and T. A. Hamor, Observations on the Synthesis and Chemistry of a Complete Series of Phenylazophenyl (C, N′) Tellurium (II) Halides (Fluoride, Chloride, Bromide and Iodide), *J. Organomet. Chem.* **1997**, *549*, 257–262.

Monoclinic, $I2/a$, a = 20.217(10), b = 4.088(3), c = 28.863(14) Å, β = 102.84(2)°, V = 2325.8(2) Å, Z' = 1, T = r.t.

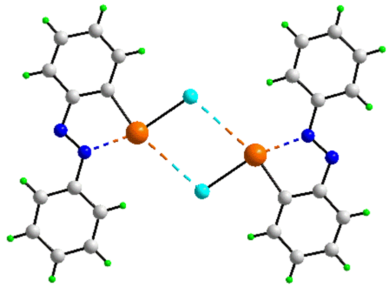

Te···Cl = 3.680(4) Å; %(d/vdW) = 96.6%; C–Te···Cl = 145.9(3)°

Te···N = 2.211(7) Å; %(d/vdW) = 61.3%; Cl–Te···N = 165.91(19)°

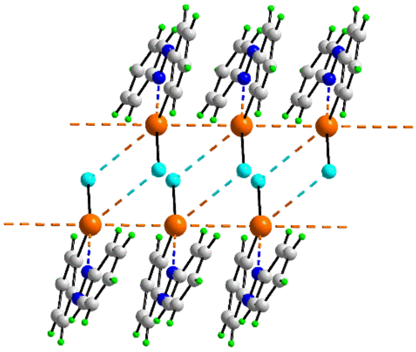

{Molecules, each with an intramolecular Te···N contact [2.211(7) Å], associate over an inversion center and feature Te···Cl interactions (shown as orange/cyan dashed lines) analogous to the iodido (**POYGIT**) and bromido (**POYGEP**) derivatives. The Two-molecule aggregates assemble into a chain and feature linear strings of Te···Te interactions [4.088(3) Å], longer intra-chain Te···N [3.968(8) Å] interactions are evident}

PAZPTE 2-phenylazophenyl-chlorido-tellurium(II) – triclinic polymorph

Cobbledick, R. E.; Einstein, F. W. B.; McWhinnie, W. R.; Musa, F. H. Some New Organotellurium Compounds Derived from Azobenzene: the Crystal and Molecular Structure of (2-Phenylazophenyl-CN')-tellurium(II) chloride. *J. Chem. Res.* **1979**, *145*, 1901–1904.

Triclinic, $P\bar{1}$, a = 9.376(6), b = 11.504(7), c = 12.616(9) Å, α = 105.73(4), β = 85.83(5), γ = 115.82(3)°, V = 1177.4(14) Å, Z' = 2, T = r.t.

Hydrogen atoms for **PAZPTE** were included in the model employing Mercury.

Macrae, C. F.; Sovago, I.; Cottrell, S. J.; Galek, P. T. A.; McCabe, P.; Pidcock, E.; Platings, M.; Shields, G. P.; Stevens, J. S.; Towler M.; Wood, P. A. (2020). Mercury 4.0: from visualization to analysis, design and prediction. *J. Appl. Crystallogr.* **2020**, *53*, 226–235.

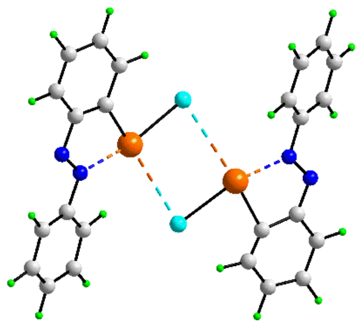

Te···Cl = 3.663(2) Å; %(d/vdW) = 96.1%; C–Te···Cl = 171.27(4)°

Te···N = 2.2257(16) Å; %(d/vdW) = 61.7%; Cl–Te···N = 167.60(5)°

second independent molecule – right-hand molecule

Te···Cl = 3.674(2) Å; %(d/vdW) = 96.4%; C–Te···Cl = 173.51(4)° { }

Te···N = 2.1926(16) Å; %(d/vdW) = 60.7%; Cl–Te···N = 165.74(5)°

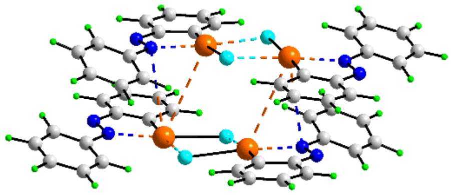

{Two independent molecules, each with an intramolecular Te···N contact [2.2257(16) & 2.1926(16) Å], associate into a non-symmetric dimer, featuring Te···Cl interactions (shown as orange/cyan dashed lines). The dimers assemble over an inversion center with long Te···Te [4.171(3) Å] and Te···N [3.743(3) Å] interactions (orange dashed lines), all longer than the sum of the respective van der Waals radii, between them. There is no Cl···Cl contact < 4.4 Å and no Cl···N contact < 4.4 Å}

COQTUX iodido-(2-(phenylazo)phenyl)selenium

Majeed, Z.; McWhinnie, W. R.; Lowe, P. R. A T-shaped Selenenyl Halide, *Acta Crystallogr., Sect. C: Struct. Chem*. **2000**, *56*, e105–e106.

Data taken from **COQTUX01** (for comparison with related structures) determined at 200 K, i.e.,

COQTUX01

Srivastava, K.; Chakraborty, T.; Singh, H. B.; Butcher, R. J. Intramolecularly Coordinated Azobenzene Selenium Derivatives: Effect of Strength of the Se···N Intramolecular Interaction on Luminescence, *Dalton Trans*. **2011**, *40*, 4489–4496.

Monoclinic, $P2_1/n$, a = 4.7893(2), b = 18.4538(10), c = 13.7159(9) Å, β = 91.237(6)°, V = 1211.94(11) Å, Z' = 1, T = 200 K.

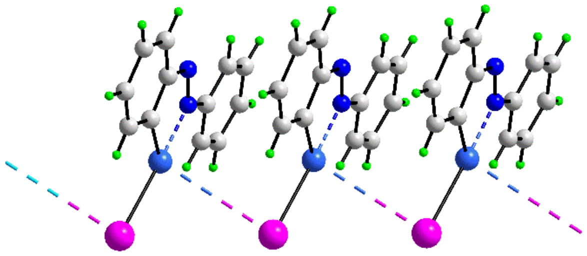

Se···I = 3.9982(7) Å; %(d/vdW) = 103.1%; I–Se···I = 87.339(16)°; C–Se···I = 121.45(17)°; N–Se···I = 95.05(13)°

Se···N = 2.053(5) Å; %(d/vdW) = 59.5%; I–Se···N = 176.39(14)°

cf. r.t. data:

Se···I = 4.0130(15) Å; %(d/vdW) = 103.4%; I–Se···I = 88.63(3)°; C–Se···I = 121.0(2)°; N–Se···I = 94.20(16)°

Se···N = 2.052(5) Å; %(d/vdW) = 59.5%; I–Se···N = 176.17(16)°

{A linear chain features a zigzag pattern of long Se···I interactions (shown as pink/light blue dashed lines) with an intramolecular Se···N contact [2.052(6) Å]; the repeat distance corresponds to the unit-cell edge, a. The closest intermolecular Se···N contact is 4.17 Å, there is no I···I contact < 4.8 Å and there is no I···N contact < 4.8 Å}

ERUVUJ bromido-(2-(phenylazo)phenyl)-selenium

Srivastava, K.; Chakraborty, T.; Singh, H. B.; Butcher, R. J. Intramolecularly Coordinated Azobenzene Selenium Derivatives: Effect of Strength of the Se···N Intramolecular Interaction on Luminescence, *Dalton Trans*. **2011**, *40*, 4489–4496.

Monoclinic, $P2_1/n$, a = 5.0040(3), b = 15.4165(8), c = 15.0856(5) Å, β = 90.843(3)°, V = 1163.64(10) Å, Z' = 1, T = 200 K.

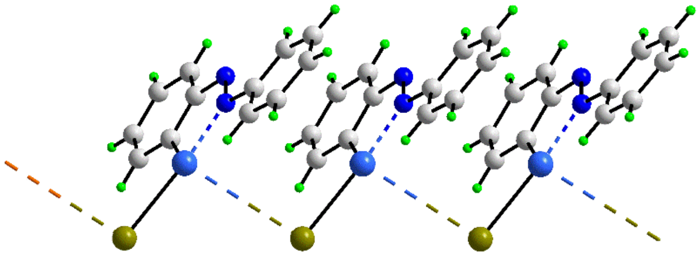

Se···Br = 3.9413(4) Å; %(d/vdW) = 105.1%; Br–Se···Br = 96.571(10)°; C–Se···Br = 121.65(8)°; N–Se···Br = 88.31(6)°

Se···N = 2.052(2) Å; %(d/vdW) = 59.5%; Br–Se···N = 174.73(6)°

{A linear chain with a zigzag feature a zigzag sequence of long Se···Br interactions (shown as dark yellow/light blue dashed lines) with an intramolecular Se···N contact [2.025(2) Å]; the repeat distance corresponds to the unit-cell edge, *a*. The closest intermolecular Se···N contact is 4.21 Å, there is no Br···Br contact < 5.0 Å and the closest Br···N contact = 4.36 Å}

ERUWIY chlorido(2-(phenylazo)phenyl)selenium

Srivastava, K.; Chakraborty, T.; Singh, H. B.; Butcher, R. J. Intramolecularly Coordinated Azobenzene Selenium Derivatives: Effect of Strength of the Se···N Intramolecular Interaction on Luminescence, *Dalton Trans.* **2011**, *40*, 4489–4496.

Orthorhombic, *Pbca*, *a* = 21.7803(5), *b* = 7.4278(2), *c* = 28.0087(8) Å, *V* = 4531.2(2) , *Z'* = 2, *T* = 200 K.

dimer:

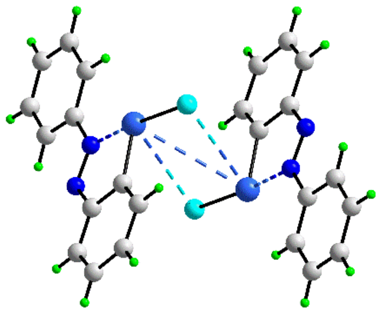

Se···Se = 3.8590(8) Å; %(d/vdW) = 101.6%; Cl–Se···Se = 72.04(4)°; C–Se···Se = 74.84(18)°; N–Se···Se = 106.80(13)°

Se···Cl = 3.9077(16) Å; %(d/vdW) = 107.1%; Cl–Se···Cl = 110.05(4)°; C–Se···Cl = 72.69(18)°; N–Se···Cl = 68.98(13)°

Se···N = 2.003(5) Å; %(d/vdW) = 53.5%; Cl–Se···N = 174.45(14)°

chain:

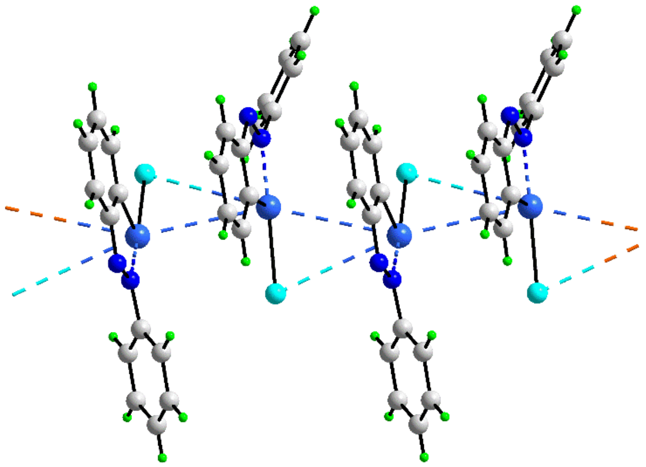

Se···Se = 3.8242(9) Å; %(d/vdW) = 100.6%; Cl–Se···Se = 72.38(5)°, C–Se···Se = 102.32(18)°; N–Se···Se = 108.24(15)°

Se···Cl = 3.881(2) Å; %(d/vdW) = 106.3%; Se–Cl···Se = 69.89(5)°; Cl–Se···Cl = 111.22(5)°, C–Se···Cl = 91.51(19)°; N–Se···Cl = 69.69(14)°

Se···N = 1.965(5) Å; %(d/vdW) = 57.0%; Cl–Se···N = 174.14(14)°

{Two independent molecules. One self-associates into a centrosymmetric dimer via a Se···Se interaction (light blue dashed lines) with supporting Se···Cl contacts; shown as light blue/cyan dashed lines). The second molecule assembles into a zigzag chain featuring Se···Se interactions with supporting Se···Cl contacts. The closest Se···N contact is 4.077(5) Å (between dimers). There are no Cl···Cl contacts < 5.0 Å. Intra-dimer Cl···N [3.697(5) Å] and intra-chain Cl···N [3.692(6) Å] contacts are apparent}

6_VOMVEY o-(3-iodopropionyl)phenyl-iodo-tellurium

Sadekov, I. D.; Ladatko, A. A.; Nivorozhkin, V. L.; Kompan, O. E.; Struchkov, Yu. T.; Minkin, V. I. Synthesis and Structure of Aromatic and Heterocyclic Tellurium Compounds. Part 29. Rearrangement of Tellurochroman-4-one 1,1-Dihalides(II) Facilitated by Intramolecular Te–O Coordination. Molecular and Crystal Structure of o-(3-Iodopropionyl) benzenetellurenyl Iodide., *Zh. Obshch. Khim.* **1990**, *60*, 2764–2769.

Monoclinic, $P2_1/c$, a = 7.694(1), b = 20.622(2), c = 7.785(1) Å, β = 95.27(1)°, V = 1230.0(3) Å^3, Z' = 1, T = r.t.

Hydrogen atoms for **VOMVEY** were included in the model employing Mercury.

Macrae, C. F.; Sovago, I.; Cottrell, S. J.; Galek, P. T. A.; McCabe, P.; Pidcock, E.; Platings, M.; Shields, G. P.; Stevens, J. S.; Towler M.; Wood, P. A. (2020). Mercury 4.0: from visualization to analysis, design and prediction. *J. Appl. Crystallogr.* **2020**, *53*, 226–235.

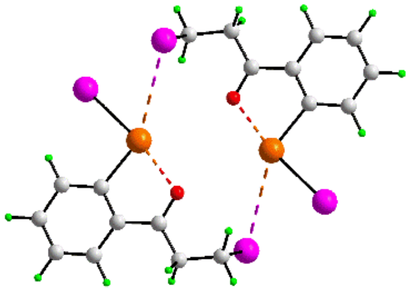

Te···I = 4.4356(10) Å; %(d/vdW) = 109.8%; I–Te···I = 80.8(2)°; C–Te···I = 134.7(2); O–Te···I = 109.10(15)°

Te···O = 2.369(6) Å; %(d/vdW) = 57.0%; I–Te···O = 169.52(15)°

{Centrosymmetric, 14-membered {···TeC_5I}$_2$ synthon with an extended, flattened chair conformation with the interacting iodine atoms being above and below the approximate plane through the remaining atoms. A close, intramolecular Te···O interaction is noted; no close intermolecular contacts involving the Te with I and O atoms}

NO CONGENERS.i

7_FUSWAS (di-iodo)-iodo-(2,6-dimethoxyphenyl)-tellurium

de Oliveira, G. M.; Faoro, E.; Lang, E. S. New Aryltellurenyl Iodides with Uncommon Valences: Synthetic and Structural Characteristics of [$RTeTeI_2R$], [R_2TeTeR_2][Te_4I_{14}], and [$RTe(I)I_2$](R= 2, 6-Dimethoxyphenyl). *Inorg. Chem.* **2009**, 48, 4607–4609.

Triclinic, $P\bar{1}$, a = 9.110(5), b = 9.529(5), c = 9.681(5) Å, α = 73.550(5), β = 80.002(5), γ = 62.359(5)°, V = 713.1(7) Å, Z' = 1, T = r.t.

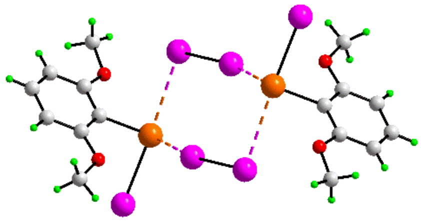

Te···I = 2.8255(14) Å; I–I···Te = 171.15(2)°

Te···I = 3.3449(19) Å; I–Te···I = 174.26(3)°

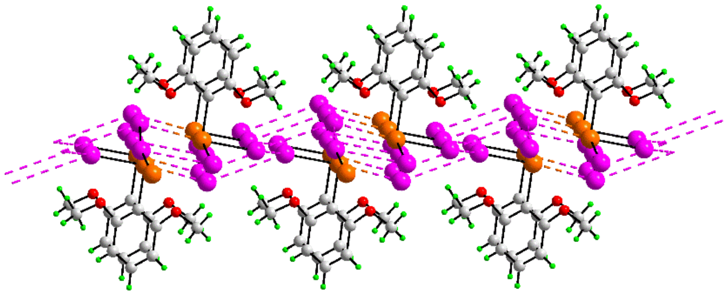

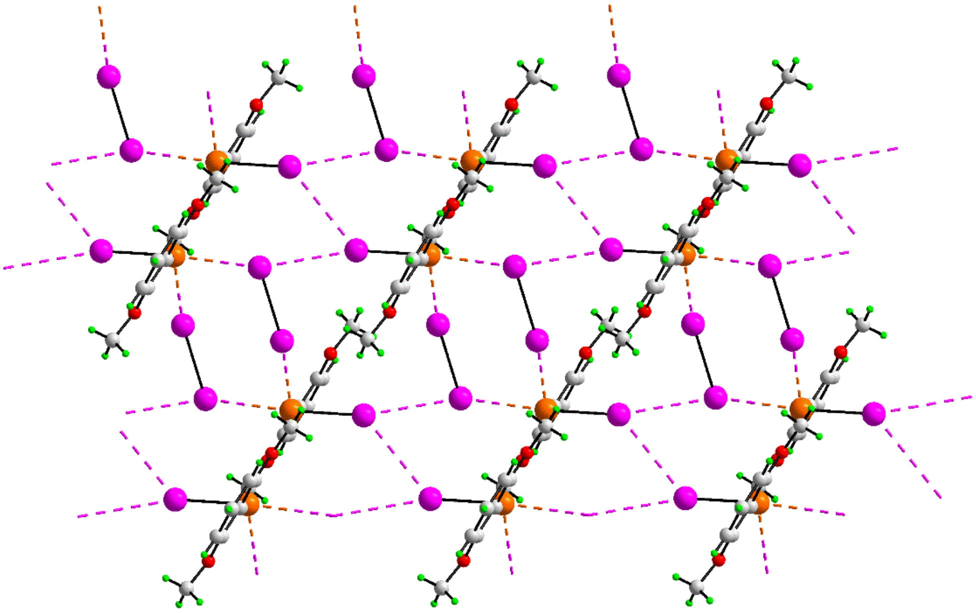

{Tellurium(II) adduct with I_2; two Te···I contacts within a centrosymmetric, six-membered {···TeI_2}$_2$ synthon; the ring is flat. Intramolecular Te···O contacts are noted with the shortest being 2.987(4) Å. Aggregates are connected into an undulating layer via long I···I [3.8921(18) and 3.9988(18) Å] interactions with Te–I···I = 147.18(2)° and 132.12(2)°, respectively}

NO CONGENERS.

8_GEDSEO iodido-(tetramethylthiourea-S)-(2-naphthyl)-tellurium(II)

Lang, E. S.; Ledesma, G. N.; Abram, U.; Vega-Teijido, M.; Caracelli, I.; Zukerman-Schpector, J. Synthesis, Crystal Structure and Theoretical Studies of Aryltellurenyl Tetramethylthiourea (tmtu) Iodine Complexes: Ph-Te(tmtu)I (1) and β-naphtyl-Te(tmtu)I (2). *Z. Kristallogr. – Cryst. Mater.* **2006**, *221*, 166–172.

Monoclinic, $P2_1/n$, a = 8.434(5), b = 11.697(5), c = 18.472(5) Å, β = 98.556(5)°, V = 1802(1) Å^3, Z' = 1, T = r.t.

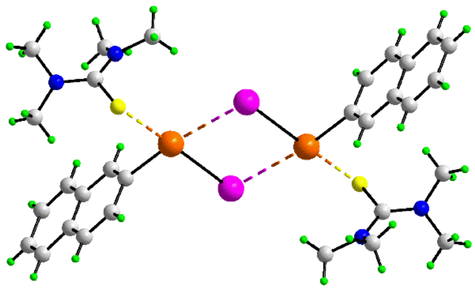

Te···I = 3.7739(14) Å; C–Te···I = 164.33(7)°

Te···S = 2.6075(9) Å; I–Te···S = 178.576(16)°

{Centrosymmetric dimer via a centrosymmetric, four-membered {···TeI}$_2$ synthon. An intermolecular Te···S contact [2.6075(9) Å] is noted}

NO CONGENERS.

9_KAJXUS iodido-thiourea-phenyl-tellurium(II)

Londero, A. J.; Pineda, N. R.; Matos, V.; Piquini, P. C.; Abram, U.; Lang, E. S. Synthesis and Characterization of Aryltellurium Compounds Including Mixed-Valence Derivatives – Evaluation of Te···S, Te···X and X···X (X= Br, I) Interactions. *J. Organomet. Chem.* **2020**, *929*, 121553.

Monoclinic, $P2_1/n$, a = 6.7354(2), b = 10.6942(4), c = 15.6336(5) Å, β = 92.995(1)°, V = 1124.55(6) Å^3, Z' = 1, T = 120 K

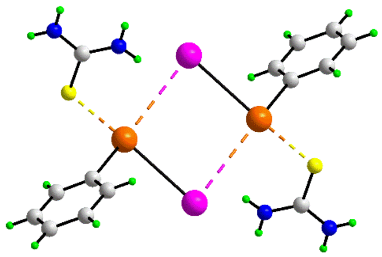

Te···I = 3.9132(3) Å; %(d/vdW) = 96.9; C–Te···I = 166.38(4)°

Te···S = 2.4919(5) Å; %(d/vdW) = 64.6; I–Te···S = 177.369(14)°

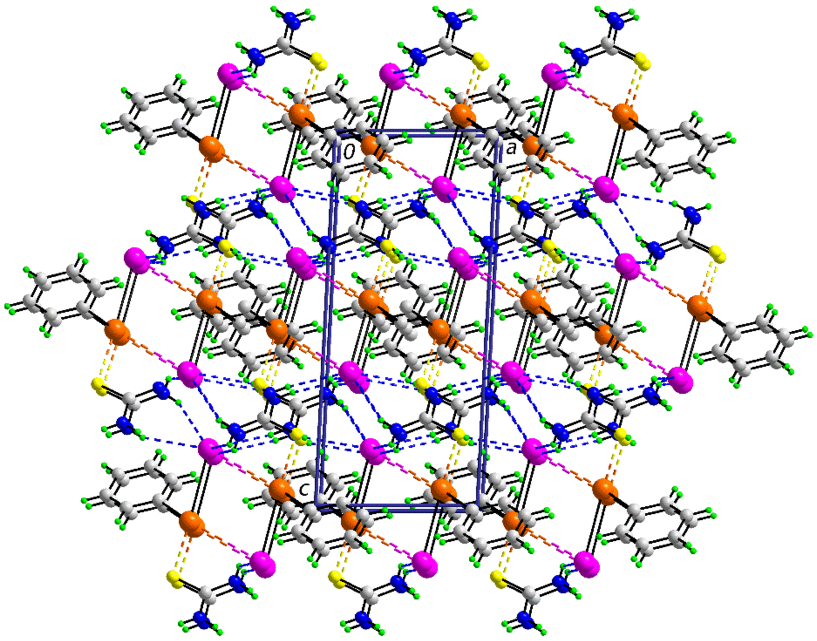

{Centrosymmetric {···TeI}$_2$ synthon. Aggregates are assembled into a three-dimensional architecture and are connected by N–H···I hydrogen bonds [2.80(3) to 2.80(3) Å] involving all thiourea-H atoms}

CONGENERS:

BZETBS: bromido-thiourea-phenyl-tellurium(II)

Foss, O.; Husebye, S. The Crystal and Molecular Structures of Complexes of Benzenetellurenyl Chloride and Bromide with Thiourea, *Acta Chem. Scand.*, 1966, **20**, 132–142.

Standard uncertainty values are not available:

Monoclinic, $P2_1/n$, a = 6.46, b = 10.73, c = 15.36 Å, β = 91.5°, V = 1064 Å^3, Z' = 1, T = r.t.

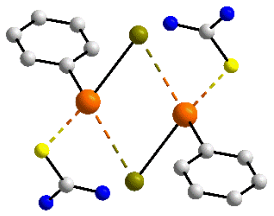

Te···Br = 3.77 Å; %(d/vdW) = 96.4; C–Te···Br = 164°

Te···S = 2.50 Å; %(d/vdW) = 64.8; Br–Te···S = 174°

{Isomorphous with **KAJXUS**. Centrosymmetric {···TeBr}$_2$ synthon; four close Br···N interactions [3.32 to 3.55 Å], representing N–H···Br hydrogen bonds occur between the two-molecule aggregated within a three-dimensional array}

BZETCS: chlorido-thiourea-phenyl-tellurium(II)

Foss, O.; Husebye, S. The Crystal and Molecular Structures of Complexes of Benzenetellurenyl Chloride and Bromide with Thiourea, *Acta Chem. Scand.*, 1966, **20**, 132–142.

Standard uncertainty values are not available:

Monoclinic, $P2_1/n$, a = 6.32, b = 10.62, c = 15.16 Å, β = 90.5°, V = 1018 Å^3, Z' = 1, T = r.t.

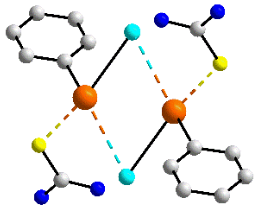

Te···Cl = 3.71 Å; %(d/vdW) = 97.4; C–Te···Cl = 164°

Te···S = 2.50 Å; %(d/vdW) = 64.8; Cl–Te···S = 172°

{Isomorphous with **KAJXUS**. Centrosymmetric {···TeCl}$_2$ synthon; four close Cl···N interactions [3.22 to 3.53 Å], representing N–H···Br hydrogen bonds occur between the two-molecule aggregated within a three-dimensional array }

10_CEMMAJ di-iodido-(triethylphosphine)tellurium(II)

Konu, J.; Chivers, T. Synthesis, Spectroscopic and Structural Characterization of Tertiary Phosphine Tellurium Dihalides Et_3PTeX_2 (X= Cl, Br, I). *Dalton Trans.* **2006**, 3941–3946.

Monoclinic, $P2_1/c$, a = 8.6469(17), b = 11.051(2), c = 13.897(3) Å, β = 104.06(3)°, V = 1288.2(5) Å^3, Z' = 1, T = 173 K

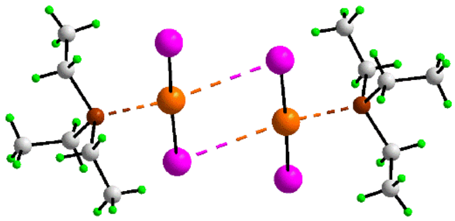

Te···I = 3.7112(12) Å; %(d/vdW) = 91.9; P···Te···I = 171.83(3)°

Te···P = 2.4899(13) Å; %(d/vdW) = 64.5; I···Te···P = 171.83(3)°

{Two-molecule aggregate located about a center of inversion; no intermolecular interactions of note}

CONGENERS:

CEMLUC: di-bromido-(triethylphosphine)tellurium(II)

Konu, J.; Chivers, T. Synthesis, Spectroscopic and Structural Characterization of Tertiary Phosphine Tellurium Dihalides Et_3PTeX_2 (X= Cl, Br, I). *Dalton Trans.* **2006**, 3941–3946.

Monoclinic, $P2_1/n$, a = 7.6660(15), b = 13.669(3), c = 11.269(2) Å, β = 90.32(3)°, V = 1180.8(4) Å^3, Z' = 1, T = 173 K

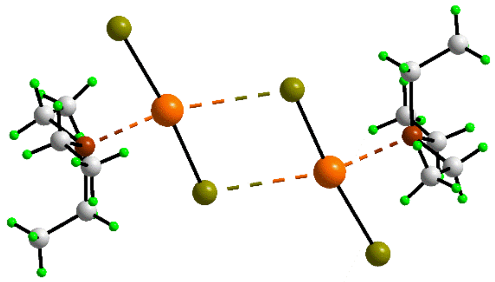

d(Te···Br) = 3.5198(7) Å; %(d/vdW) = 90.0; P–Te···Br = 161.82(3)°

Te···P = 2.4729(11) Å; %(d/vdW) = 64.1; Br···Te···P = 161.82(3)°

{Two-molecule aggregate located about a center of inversion; no intermolecular interactions of note}

CEMLOW: di-chlorido-(triethylphosphine)tellurium(II)

Konu, J.; Chivers, T. Synthesis, Spectroscopic and Structural Characterization of Tertiary Phosphine Tellurium Dihalides Et_3PTeX_2 (X= Cl, Br, I). *Dalton Trans.* **2006**, 3941–3946.

Monoclinic, $P2_1/n$, a = 7.6125(15), b = 13.301(3), c = 10.905(2) Å, β = 90.51(3)°, V = 1104.1(4) Å^3, Z' = 1, T = 173 K

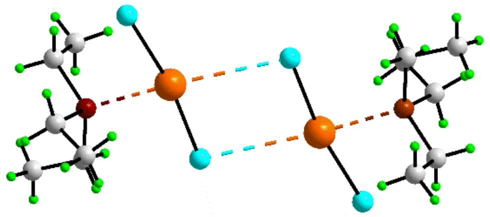

d(Te···Cl) = 3.4341(14) Å; %(d/vdW) = 90.1; P–Te···Cl = 161.59(3)°

Te···P = 2.4656(12) Å; %(d/vdW) = 63.9; Cl···Te···P = 161.59(3)°

{Two-molecule aggregate located about a center of inversion; no intermolecular interactions of note}
